# Cold and dry winter conditions are associated with greater SARS-CoV-2 transmission at regional level in western countries during the first epidemic wave

**DOI:** 10.1038/s41598-021-91798-9

**Published:** 2021-06-17

**Authors:** Jordi Landier, Juliette Paireau, Stanislas Rebaudet, Eva Legendre, Laurent Lehot, Arnaud Fontanet, Simon Cauchemez, Jean Gaudart

**Affiliations:** 1 IRD, Aix Marseille Univ, INSERM, SESSTIM, Aix Marseille Institute of Public Health, ISSPAM, Marseille, France; 2 Mathematical Modelling of Infectious Diseases Unit, Institut Pasteur, UMR2000, CNRS, Paris, France; 3 Santé publique France, French National Public Health Agency, Saint Maurice, France; 4 Hôpital Européen Marseille, Marseille, France; 5 Emerging Infectious Diseases Unit, Institut Pasteur, Paris, France; 6 PACRI Unit, Conservatoire National des Arts et Métiers, Paris, France; 7 Aix Marseille Univ, APHM, INSERM, IRD, SESSTIM, ISSPAM, Hop Timone, BioSTIC, Marseille, France

**Keywords:** Infectious diseases, Viral infection, Medical research, Epidemiology, Diseases, Respiratory tract diseases

## Abstract

Higher transmissibility of SARS-CoV-2 in cold and dry weather conditions has been hypothesized since the onset of the COVID-19 pandemic but the level of epidemiological evidence remains low. During the first wave of the pandemic, Spain, Italy, France, Portugal, Canada and USA presented an early spread, a heavy COVID-19 burden, and low initial public health response until lockdowns. In a context when testing was limited, we calculated the basic reproduction number (R_0_) in 63 regions from the growth in regional death counts. After adjusting for population density, early spread of the epidemic, and age structure, temperature and humidity were negatively associated with SARS-CoV-2 transmissibility. A reduction of mean absolute humidity by 1 g/m^3^ was associated with a 0.15-unit increase of R_0_. Below 10 °C, a temperature reduction of 1 °C was associated with a 0.16-unit increase of R_0_. Our results confirm a dependency of SARS-CoV-2 transmissibility to weather conditions in the absence of control measures during the first wave. The transition from summer to winter, corresponding to drop in temperature associated with an overall decrease in absolute humidity, likely contributed to the intensification of the second wave in north-west hemisphere countries. Non-pharmaceutical interventions must be adjusted to account for increased transmissibility in winter conditions.

## Introduction

The spread of the first wave of SARS-CoV-2 in February–March 2020 was largely heterogeneous between countries. Various determinants were proposed to explain the differential spread of the virus. Some Asian countries like Japan, South Korea, Vietnam and Thailand and some isolated countries like Australia, New Zealand, Iceland, experienced only limited transmission and stood out for high levels of preparedness and efficient response strategies^1,2^. Countries in South West Europe and North America displayed low levels of preparedness and deployed no effective response before lockdowns. Still in these countries, SARS-CoV-2 virus spread heterogeneously at the regional level, as observed from hospitalization, death counts, and confirmed by serological surveys^3,4,37^.

Epidemic spread is characterized by the basic reproduction number (R_0_). R_0_ expresses the average number of secondary cases resulting from a given case in the context of a naive population. R_0_ depends on the duration of the infectious period, the probability of infecting a susceptible individual during a direct or indirect contact, and the number of new susceptible individuals contacted per unit of time^5^. Environmental parameters affect R_0_: population density may increase the probability of contacts between individuals and weather and season can also play an important role. Most known respiratory viruses spread during the cold season in the temperate Northern hemisphere^6^. Weather conditions in winter can affect individual susceptibility to infection through irritation of the nasal mucosa. They can also influence the behaviour of individuals towards conditions prone to transmission: living or gathering in closed spaces increasing exposure, and indoors conditions (heated, dry air) favouring virus persistence or individual susceptibility^6^. In addition, temperature, humidity, and UV, might directly affect the virus survival and modify infectiousness^7–9^. SARS-CoV-2 is indeed likely subject to important environmental transmission through aerosols or fomites^10^. Individual preventive behaviours (masks), collective strategies reducing mobility and contacts (lockdowns) or limiting the duration of the infectious period (detection and isolation) modify the number of secondary cases and the effective reproduction number can be calculated, which accounts for these alterations in the “natural” history of transmission.

In spite of a large number of studies, the evidence regarding the link between weather conditions and SARS-CoV-2 transmission remains limited. A systematic review including studies conducted up to 15 May 2020 retained 61 studies analysing the relationship between COVID-19 epidemic and environmental factors^11^. Methodological issues included the lack of controlling for confounding factors such as population density^11^. Inappropriate epidemiological and statistical methods were also pointed out^11,12^. Comparison between countries with different counter epidemic responses, testing strategy, or delayed onset of the epidemic might also have led to inconsistent results^11^. An earlier review retaining 17 studies highlighted the risk of bias and the low level of available evidence^13^. Between 15 May 2020 and 15 December 2020, our systematic search identified 82 research articles, of which only 15 (18%) analysed the growth rate or the reproduction number of SARS-CoV-2 (Appendix p2). Only four studies of climate and reproduction number included adjustments for confounding factors, and three of four included relevant covariates of population mobility, population density, and took into account interventions when necessary^14–16^. Of these, two were conducted over small geographical units: one study of 212 US counties identified a negative relationship between temperature and SARS-CoV-2 case growth, while the other study including 28 Japanese prefectures identified a positive relationship^14,15^. The third study was at the global scale for 203 states (in the USA, Canada, Australia and China) or countries and identified a negative association between UV light exposure and SARS-CoV-2 case growth. Higher temperatures were associated with slower growth only after adjustment for UV light exposure^16^. All three studies identified a positive association between population density and epidemic growth.

Overall, multiple studies described a negative relationship between temperature and COVID-19 outcomes but the majority were unadjusted ecological correlation studies with strong risk of bias bringing low quality evidence^11^, and evidence from multivariable growth studies remains ambiguous.

A modelling study across five continents defined the range of possible dependency between SARS-CoV-2 transmission and absolute humidity (AH), based on two known coronaviruses and influenza^17^, and recent models still do not include specific SARS-CoV-2 data^18^. More precise estimations of the effect of meteorological conditions on the spread of SARS-CoV-2 are required to better anticipate and inform policies regarding seasonal adjustments^19^.

The objective of this study was to evaluate the contribution of weather parameters in the transmission of SARS-CoV-2, by analysing their effect on SARS-CoV-2 R_0_ in a context of low public health response during the early phase of the first wave in six north-western hemisphere countries.

## Results

### Region selection

The six countries (USA, Canada, Spain, Italy, France and Portugal) included 128 regions or states. After applying exclusion criteria, the analysis was performed over 63 regions with sufficient exponential growth and complete weather data (Fig. 1, Figure S1): 24 states in the USA, two states in Canada, 11 regions in France, 12 autonomous communities in Spain, 13 regions in Italy and one region in Portugal (hereafter ‘regions’).

**Figu﻿re 1:**
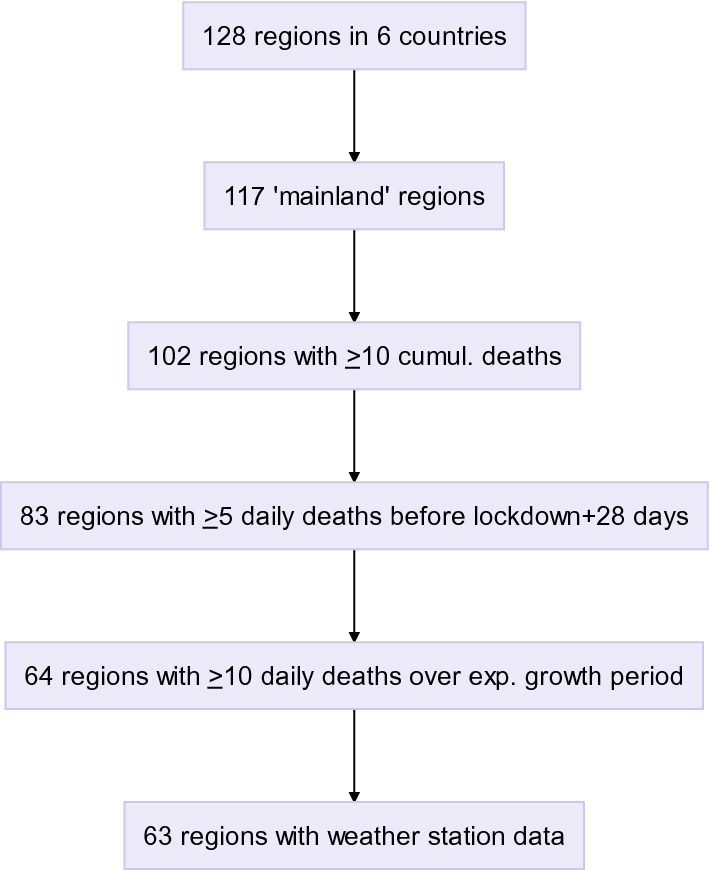
Study flow chart.

### Data description

**Figure 2:**
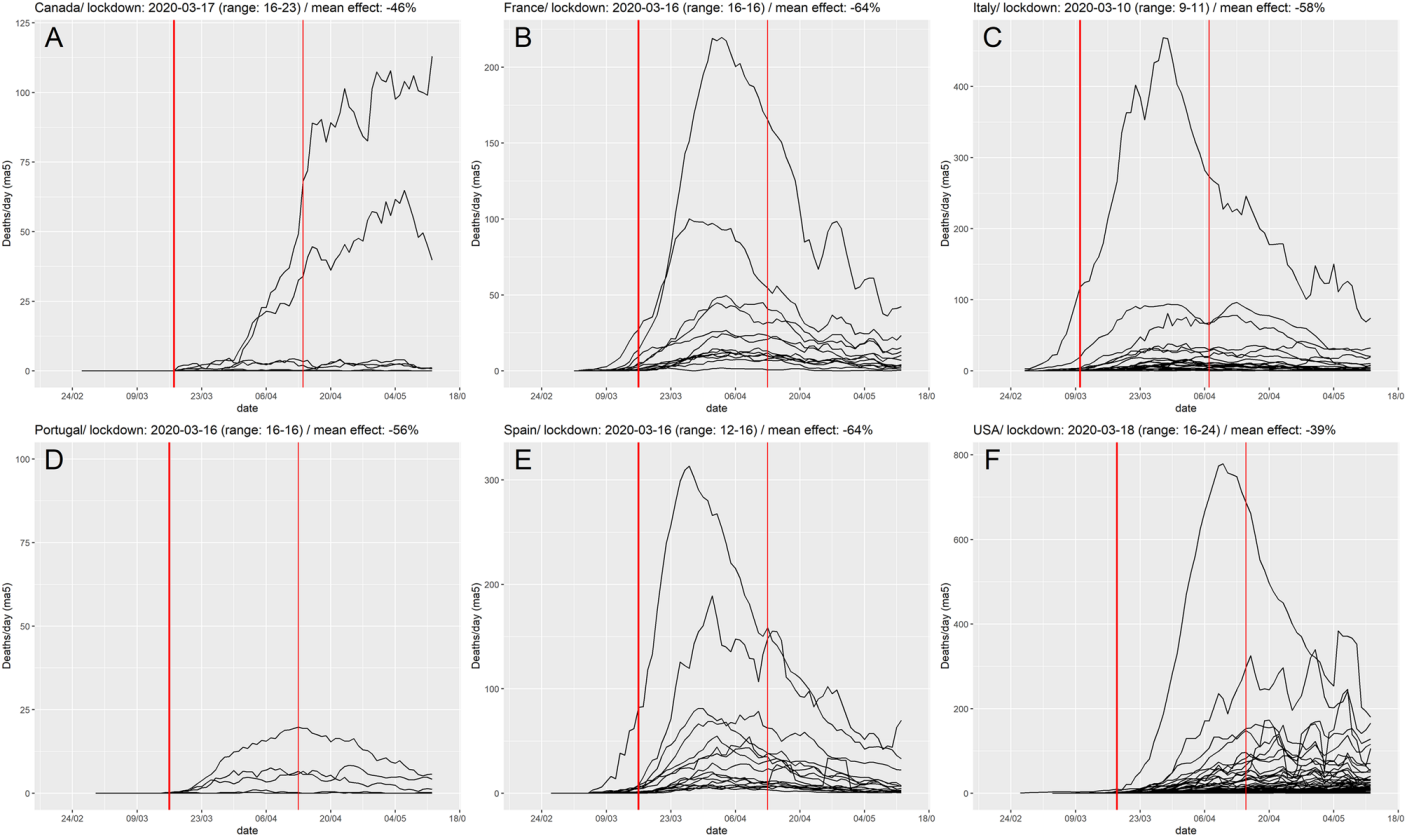
Deaths per day, by region and by country. The thick red line figures the median date of lockdown by each country, and the thin red line the median date + 28 days.

The maximal daily death count was 39 deaths/day in median (interquartile range (IQR) = 18–83, max = 779) and occurred 25 days (median; IQR = 19–43) after the start of the lockdown (Fig. 2). Of note, larger reductions in human mobility patterns after lockdown as assessed from Google mobility led to shorter delay (Spearman correlation coefficient = 0.63, *p* < 0.0001, Figure S3). The delay between the start of lockdown and the peak in daily death count was reduced to 19 days (median; IQR = 17–27) for South European countries with nationwide lockdowns and strong mobility reductions (Italy, France, Spain and Portugal, > 60% in average, Figure S3).

R_0_ was estimated from death counts over an exponential growth period that had a median duration of 11 days (IQR = 9–14, range = 5–19). The R_0_ estimation period started 5 days (median across regions; IQR = 0–8.5) and ended 16 days after the date of lockdown (IQR = 11–21, range 1–27). Figure S4 presents detailed calculation periods for all regions.

**Figure 3:**
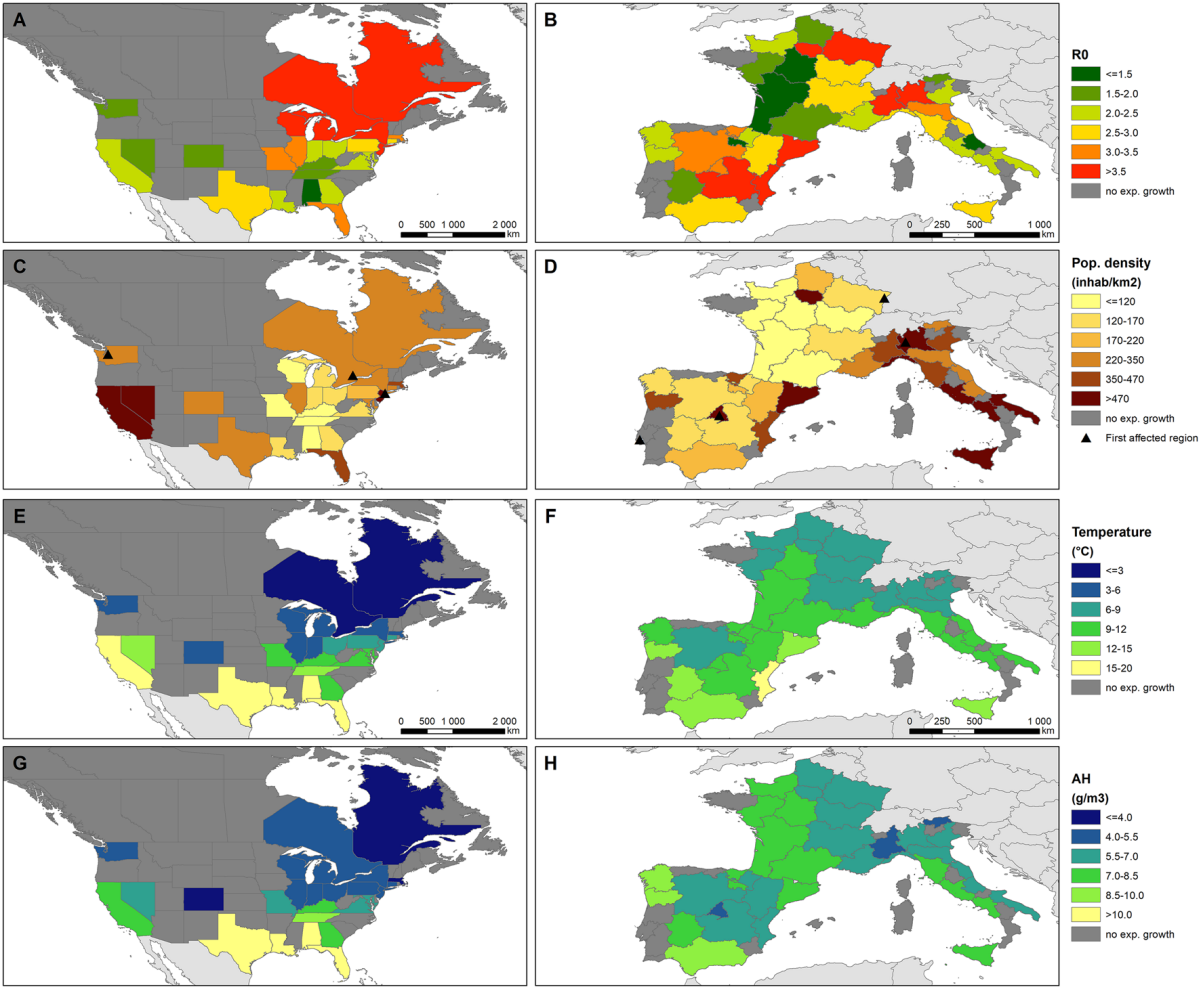
Map of regional values for R_0_ and selected covariates, panels are presented by continent. (**A**, **B**) R_0_, (**C**, **D**) population density (inhabitants per km^2^) and first region affected with 10 cumulative deaths, (**D**, **E**) mean temperature, (**F**, **G**) mean absolute humidity (AH). Map generated using ArcGIS v10.7.1 (ESRI, Redlands, CA).

The median R_0_ value was 2.58 (IQR = 2.08–2.66). R_0_ estimates were lowest (< 1.5) in two regions in France and one in Spain, the USA and Italy (respectively in Centre-Val de Loire, Nouvelle Aquitaine, La Rioja, Alabama and Abruzzo). R_0_ was highest (> 4.0) in New York (USA), Lombardia and Piemonte (Italy), Castilla-La Mancha (Spain) and Ontario (Canada) (Fig. 3A,B). R_0_ values exhibited significant spatial autocorrelation (Moran’s I = 0.20, *p* = 0.0087).

**Figure 4:**
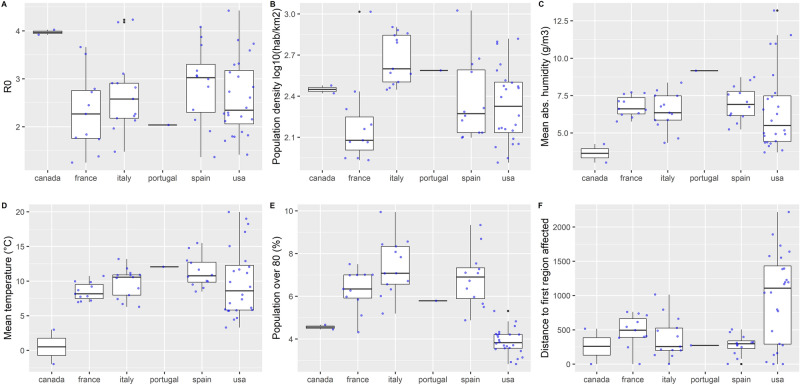
Distribution of R_0_ and selected covariates by country. (**A**) R_0_, (**B**) population density (log10 inhabitants/km^2^). (**C**) Mean AH (g/m^3^). (**D**) Mean temperature (°C). (**E**) Population over 80 years old (%), (**F**) distance to the first region affected (km). The box represents the interquartile range and the median; whiskers correspond to the minimum between highest value and 1.5 IQR; black dots to outliers. All observations are ploted in light grey.

Demographic and climatic variables were heterogeneous between countries (Figs. 3, 4). Regional population density (excluding areas with < 5 inhabitants/km^2^) ranged between 82 and 1056 inhabitants/km^2^ (median = 271, Fig. 3C,D) and was overall higher in Italy (Fig. 4B). Weather covariates were introduced as mean, minimum or maximum value over the estimated transmission period. Across the 63 regions, mean AH during the transmission period was 4.98 g/m^3^ in median (IQR = 4.29–5.99, range = 2.26–11.32, Fig. 3E,F) corresponding to a median dew point temperature (DP) of 3.7 °C (IQR = 0.9–6.3, range =  − 7.7 to + 15.3). Mean temperature was 9.8 °C in median (IQR = 7.1–11.5, range =  − 2.0–19.9). Distance to the first region with 10 cumulative deaths was 406 km in median (IQR = 228–794) and much larger in the USA compared to European countries (Fig. 4F).

### Factors associated with R_0_

**Figure 5:**
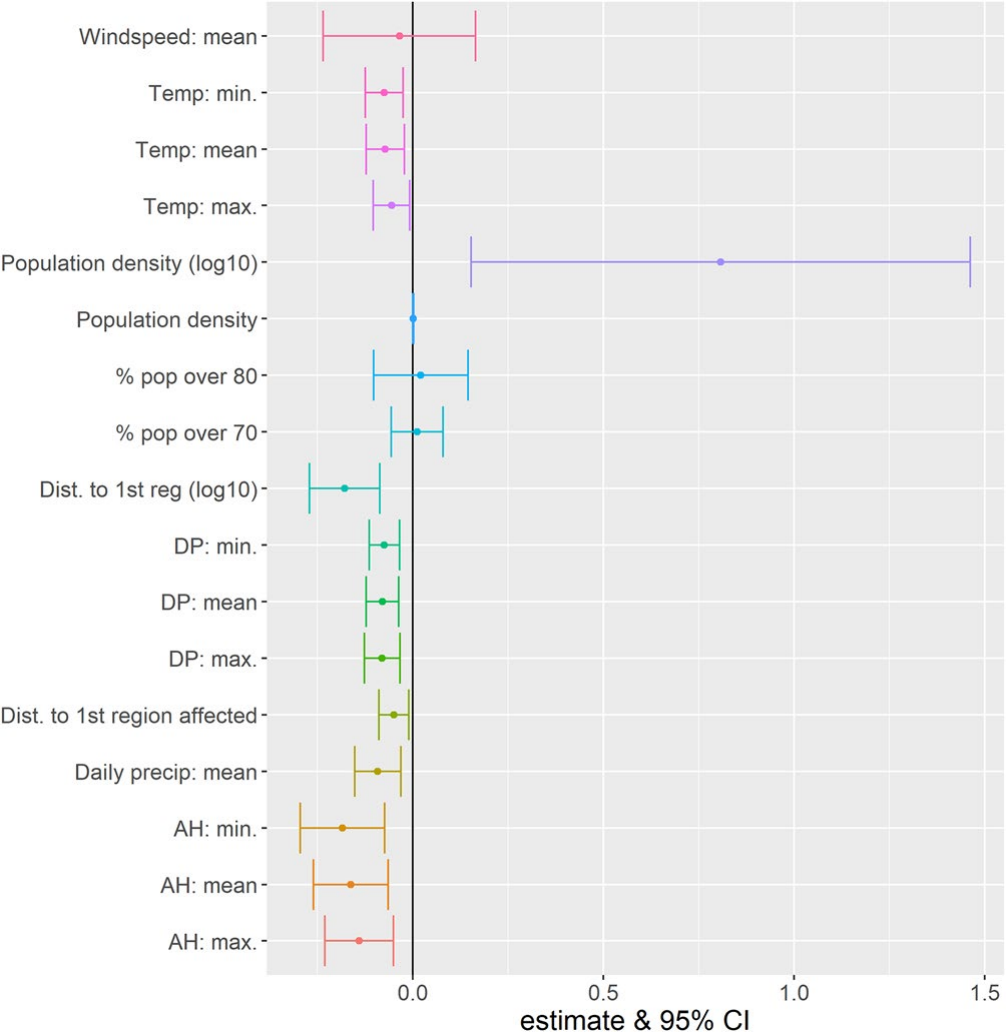
Change in R_0_ on an additive scale estimated from the univariable model assuming a linear relationship between R_0_ and the different variables over their entire range.

Each variable was included in a univariate model assuming linear (Fig. 5, Table S2) or non-linear (spline smoothing, Table S3) relationships with R_0_. We identified significant relationships between R_0_ value and temperature, AH or DP, average cumulative precipitation per day, but not with mean wind speed over the transmission period.

Multivariable models were constructed according to a directed acyclic graph (Figure S2). After adjustment for distance to the first affected region in the country, percentage of population over 80 and population density, there was a consistent relationship between increasing temperature, AH or DP, and decreasing R_0_. No relationship was found with average cumulative precipitation per day.

**Figure 6:**
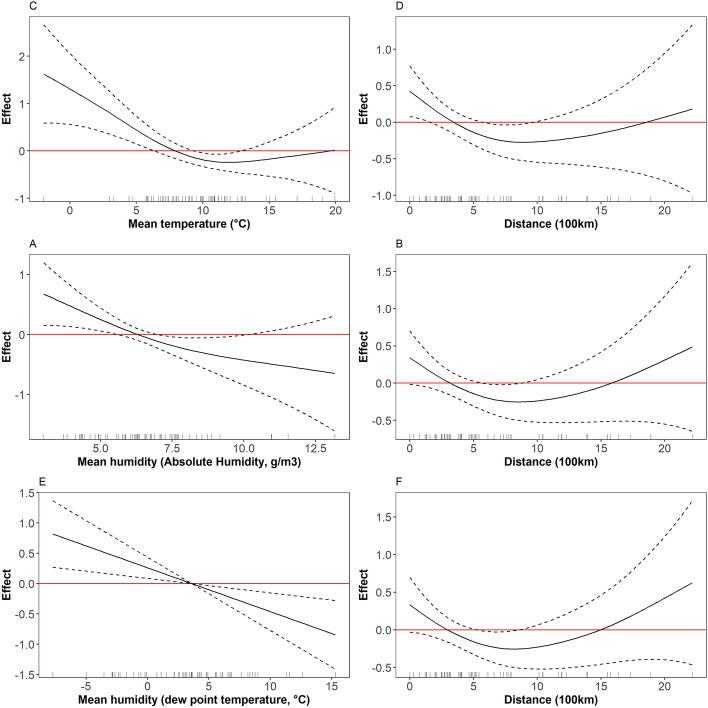
Non-linear effects for weather parameters obtained in the multivariable model for R_0_ analysis (see Table 1 for linear effects). (**A**) Temperature, model 1. (**B**) Distance to first region affected, model 1. (**C**) AH, model 2. (**D**) Distance to first region affected, model 2. (**E**) DP, model 3. (**F**) Distance to first region affected, model 3.

**Figure 7:**
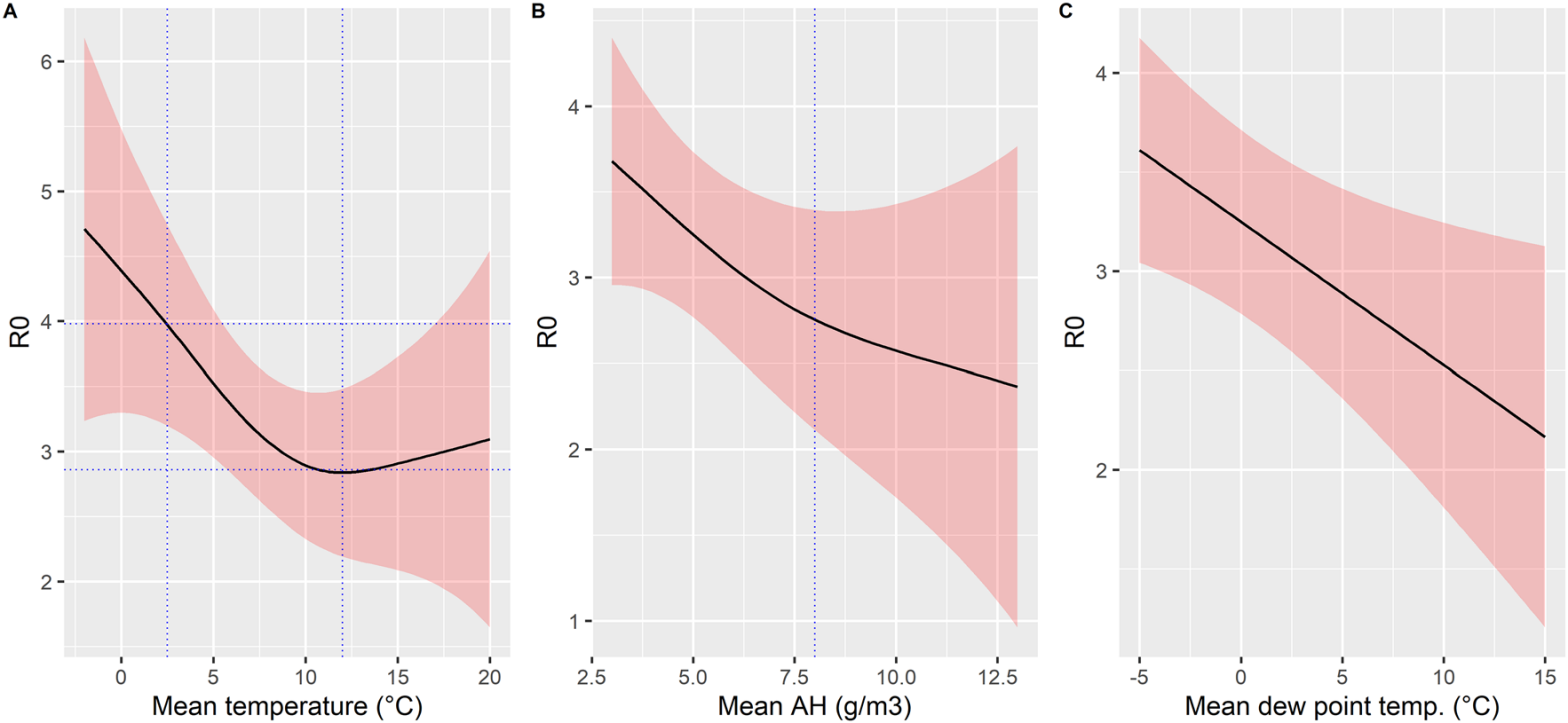
Summary of estimated effect of temperature (**A**), AH (**B**) or DP (**C**) on R_0_ assuming a region with average population density (248 persons/km^2^) and percentage of inhabitants > 80 years (5.6%), and corresponding to the first region first affected (distance = 0 km).

Mean temperature led to the model with the largest deviance explained compared to models including minimum or maximum temperature, or any AH or DP measurements (Table 1). In this model, a tenfold (+ 1 log10 unit) increase in population density was associated with a + 0.67 R_0_ unit increase (95% Confidence Interval (95%CI) = 0.05–1.28). The proportion of population aged 80 years and older was not significantly associated with R_0_. The relationship between mean temperature and R_0_ was not linear. A strong, nearly linear drop of approximately 1.0 R_0_ unit was observed between 2.5 and 10 °C, and a plateau beyond 10 °C (Figs. 6, 7). The residuals from this model did not exhibit significant spatial autocorrelation (Moran’s I = 0.054, *p* = 0.215).

**Table 1:** Multivariable model results for the relationship between R_0_ and weather parameters, obtained with the hierarchical generalized additive model.

Model	Variable	Estimate	95%CI	p-value
**Model 1**	Intercept	0.78	[− 0.88–2.45]	0.36152
	Population density (log10)	0.67	[0.07–1.26]	0.03218
	% population over 80	0.05	[− 0.08–0.18]	0.44470
	Distance to first region affected in the country/coast	**spline**		0.08007
	**Mean temperature**	**spline**		0.00655
	Dev. explained: 41.5%			
**Model 2**	Intercept	1.28	[− 0.36–2.92]	0.13239
	Population density (log10)	0.50	[− 0.11–1.11]	0.11294
	% population over 80	0.03	[− 0.1–0.16]	0.61862
	Distance to first region affected in the country/coast	**spline**		0.115
	**Mean AH**	**spline**		0.03401
	Dev. explained: 33.6%			
**Model 3**	Intercept	1.20	[− 0.47–2.88]	0.16427
	Population density (log10)	0.49	[− 0.12–1.1]	0.12005
	% population over 80	0.05	[− 0.08–0.18]	0.47092
	Distance to first region affected in the country/coast	**spline**		0.09756
	**Mean dew point temperature**	**spline**		0.00494
	Dev. explained: 34.6%			

Weather parameters are temperature, AH, and DP temperature, adjusted for distance to the first region affected, population density, and elderly population. Non-linear effects are presented in Fig. 6. Models assuming linear effects for weather covariates are presented in Table S4.

Mean AH and mean DP values exhibited similar profiles with increasing values associated with decreasing R_0_ values (Table 1, Fig. 6). It seemed that the relationship between R_0_ and AH or DP did not reach a plateau. Assuming a linear relationship, a 1 g/m^3^ higher AH translated in a 0.15 unit lower regional R_0_, and a 1 °C higher DP translated in a 0.08 unit lower R_0_ (Table S4). The residuals from these model did not exhibit significant spatial autocorrelation (Moran’s I = 0.096, *p* = 0.105 for AH, Moran’s I = 0.0942, *p* = 0.11 for DP).

### Sensitivity analyses

In order to evaluate the importance of the 3-week delay between meteorological variables measurement (during estimated infection period) and R_0_ calculation (period when corresponding deaths occurred) we studied the relationship between R_0_ and meteorological variables using different delays, from 2 weeks (lag =  − 1) to 8 weeks (lag = 5), with the 3-week delay used in the main analysis corresponding to the reference value (lag = 0). Using lagged weather summary values as linear univariate predictors, our statistical model found similar relationships between R_0_ and temperature variables, but 0-, 1- and 5-week lags led to the strongest effect for mean AH and mean DP (Figure S5). Using lagged weather summary values as non-linear predictors in the multivariate model, the shapes of the relationships between temperature, AH and DP remained similar, with a nearly linear drop reaching a plateau at values corresponding to milder winter weather/climate (Figure S6). Overall, correlations were strong between weather summary observations at the different lags (Figure S7). Correlations of lagged temperature and humidity observations was highest between − 1, 0, 1 and 5-week lagged observations compared to other lags.

When setting the upper limit of the R_0_ calculation window to 18 days after date of lockdown, 10 additional regions were excluded because they failed to meet the 10 daily deaths limit over the narrower R_0_ calculation window and the analysis was conducted on 53 regions. After adjusting for population density, population over 80 and distance to the first region affected, the non-linear relationship between temperature and R_0_ remained unchanged, reaching a plateau around 10 °C (spline *p* value = 0.0475). The shape of the relationship between AH, respectively DP, and R_0_ remained similar but did not reach statistical significance (*p* value = 0.19). Later AH or DP summary values, corresponding to 1 week later than the estimated transmission period (i.e. 2-week lag from R_0_ calculation period), appeared to restore the relationship with R_0_ (*p* = 0.0661, respectively *p* = 0.0667), although not reaching statistical significance threshold.

Fitting continent-specific splines also resulted in low statistical power: only 37 regions remained in South Europe and 26 in North America. After adjusting for population density, population over 80 and distance to the first region affected, increasing humidity variables (AH or DP) were associated with a decreasing R_0_ (continent-specific spline *p* values were 0.0915 (North America) and 0.0988 (South Europe) for AH, respectively 0.0506 and 0.1078 for DP, not reaching statistical significance). The relationship between R_0_ and mean temperature was markedly different between North America (temperature increase associated with R_0_ decrease, *p* = 0.005) and South Europe (*p* = 0.17). Assuming continent-specific effects of the distance to the first region affected on R_0_ indicated that distance played a significant role in South Europe, but not in North America, without modifying the effect of temperature, AH or DP on R_0_.

## Discussion

In this study, we analysed SARS-CoV-2 propagation parameter R_0_ during the first wave of the pandemic in 63 regions of six north western hemisphere countries. We showed that R_0_ values were influenced by population density (+ 0.6 for a tenfold increase in density), by proximity with the first epidemic focus of the country or coast for USA (− 0.3 for a tenfold increase in distance to the first region to record 10 COVID-19 deaths), and by weather or climate conditions. For regions with mean temperatures below 10 °C during the transmission period, a linear association was observed with R_0_ values: a 1 °C increase in temperature between regions was associated with a 0.16-unit decrease in R_0_. A 1 g/m^3^ increase in mean AH was associated with a 0.15-unit decrease in R_0_ (Figure 7). Similar results were obtained with DP, with a 1 °C increase associated with a 0.08-unit decrease in R_0_ (Table S4). After adjusting for major confounders and spatial autocorrelation, our results indicate that weather conditions brought a significant contribution to drive the magnitude of the first wave, even if it was limited by an initial heterogeneous spread of the virus, which protected regions located furthest away from the first foci.

This study relied on a regional scale analysis and accounted for different dynamics within the same country. The overall epidemic wave at country level was actually the sum of diverse dynamics, as is obvious for large countries but also true for Spain, Italy or France, where regions were heterogeneously affected, as confirmed by serological studies^3,20^. Likewise, weather or climate heterogeneity between regions of a given country was large. By analyzing distinct spatial units with heterogeneous population density and weather, we were able to assess the effect of parameters that may be otherwise confounded by country-level parameters such as response strategy, timing of the analysis period compared to the progression of the epidemic, but also age structure of the population.

The six countries were selected due to their homogeneous location and the low efficacy of their early counter epidemic responses. We are therefore as close as possible of conditions allowing R_0_ estimation. This four-parameter hierarchical generalized additive model provides an explanation of up to 42% of R_0_ variability across 63 affected regions of the northern hemisphere, thus providing useful insights on the drivers of the first wave, and allowing to estimate the contribution of population density and weather conditions for the next waves, when the effect of local introduction is no longer relevant.

We analyzed R_0_, a parameter derived from the exponential growth rate, which is more robust to variations in reporting standards between countries or regions and requires less stringent assumptions on reporting standards comparability. It is also less biased compared to indicators based on cumulative counts or cumulative incidence rates. Indeed, the cumulative death (or case) count in a region depends on: the duration since the start of the epidemic in the region, the basic reproduction number (subsequently the effective reproduction number) and the number of seeding events: regions were not affected simultaneously, and their numbers of seeding events were likely different (but nearly impossible to quantify). The definition of a time period over which to sum counts to fully account for the epidemic requires many assumptions. In order to try and quantify the effect of climate using case/death counts, it would be necessary to account for factors influencing R_0_ as we did here, but also for factors influencing the number of seeding events and the duration of the epidemic (such as the date of introduction, the intensity of lockdown…). Our  conservative assumptions excluded regions without a clearly defined exponential growth phase from this analysis, using an arbitrary 10 deaths threshold (for starting at 10 cumulative deaths and for exponential growth phase at 10 daily deaths). Given what is known of the case-fatality ratio, this threshold corresponded to >1000 infections. This may have excluded regions with a lower growth, but identifying a relationship between R_0_ and temperature, AH or DP on the regions with the highest R_0_ remains relevant irrespectively of the relationship in low growth regions.

We acknowledge several limits. The first is the necessity to rely on death counts to estimate R_0_ during this wave due to the lack of information on actual infections from limited testing and the lack of consistency between countries for hospitalization counts. This assumption is however similar to usual assumptions for R_0_ estimation based on diagnosed cases, since true number of infections remains unknown and detection occurs at variable delays after infection.

A second limit is the use of a single summary weather observation over the assumed transmission period, which may fail to express the dynamical aspects of weather. The choice of fixed, 1-week increments to study the effect variations in the weather summary window may be too coarse to capture effects for the narrowest exponential growth periods. The sensitivity analysis showed a slight increase in effect when considering weather summary values calculated one week earlier than the estimated transmission period, but the 18-day sensitivity analysis showed a stronger negative relationship between R_0_ and AH summary values occuring 1-week later than the estimated transmission period. This study could also only evaluate the effect of a limited range of weather conditions on SARS-CoV-2 transmission, since the number of observations with a mean temperature below 2.5 °C or above 15 °C was low. This prevents from analyzing the effect of higher temperatures such as during autumn. This restriction was however necessary to achieve a minimal homogeneity of the studied regions in terms of their exposure and response to the pandemic. Finally, this analysis includes only 63 regions and may lack statistical power. The necessity to ensure that the epidemic growth of deaths was sufficient led to the exclusion of regions that may be affected, but not enough for daily deaths counts to reach 10 deaths/day.

Finally, this study showed an association between SARS-CoV-2 R_0_ and temperature/AH, but due to the strong correlation between AH and temperature in the seasonal conditions analysed here, it is difficult to determine which parameter is more important and they could not be analysed in combination^21^. The continent-specific analysis suggests that the relationship between AH and R_0_ was more stable than that of temperature, which was strong in the US but less so in South Europe. We could not conclude whether the relationship results from a direct role of weather on individual susceptibility to viral invasion (e.g. dry nasal mucosa from indoors heating and outdoors cold) or on viral persistence/survival, or from an indirect role of climate on human behaviors (e.g. regions with cold winter favor more indoors living conditions and lead to bigger infection opportunities). Interestingly, relative humidity and temperature levels associated with experimental decrease in virus survival corresponded to the range of AH values associated with R_0_ decrease in this study (Figure S9)^8^.

UV irradiance was identified as an important parameter to inactivate viral particles and could thus also bring an important contribution to limiting viral transmission. UV data was not included in reported data from this weather-station network. However, strong correlations exist between temperature, latitude and UV irradiance, which would be difficult to disentangle in an aggregated analysis. If warmer, more humid winter conditions directly influence transmission by limiting virus survival, it is possible that part of this effect originates from higher UV irradiance.

## Conclusion

Our study shows an important dependency of SARS-CoV-2 transmission to weather/climate, with each 1°C reduction in mean regional temperature below 10°C leading to a 0.16-unit increase in R_0_ , or each 1 g/m^3^ reduction in AH leading to a 0.15-unit increase in R_0_. Northern hemisphere countries experienced a new wave of SARS-CoV-2 infections during autumnal transition from summer to winter (colder temperatures and lower absolute humidity levels), while still actively maintaining control strategies^22^. When planning to adjust the level of restrictions on social activities, public health strategies need to account for the increased transmissibility of SARS-CoV-2 when/where cold and dry winter conditions are prevalent.

## Material and methods

### Study design

In order to compare the drivers of the epidemic dynamics between regions accurately, we calculated the R_0_ of the virus for each region affected by the first wave of COVID-19 epidemic in six countries, using the dynamics of the daily death counts. The study focused on the four countries hit earliest in Europe and North America (Italy, Spain, France and USA). Canada and Portugal provided geographical contiguity. These six countries shared similar location in the western part of the northern hemisphere, approximately between 25 and 50° of latitude (Figure S1) defining winter conditions at the start of the first wave of the epidemic. Furthermore, they also all underwent significant SARS-CoV-2 transmission in a context of low public health response.

This analysis was conducted at the first administrative subdivision of the countries, corresponding to states in USA and Canada, autonomous communities in Spain, and regions in Italy (regioni), Portugal (região) and France (regions), all referred to as “region” in this study.

Confirmed case counts were insufficiently reliable due to overall lack of tests and different testing strategies between countries, between regions of a given country and between periods for a given region. Hospitalization counts were not available consistently at regional level across countries. Overall, COVID-19 deaths were preferred as they were less likely to have different definitions within the same region or country and to undergo significant changes in their definition over the study period. R_0_ is an indicator of the speed of progression of the outbreak, and was therefore less likely to be biased compared to indicators based on cumulative counts or cumulative incidence rates.

Estimating R_0_ values based on deaths counts relies on the minimal assumption that the infection-fatality rate was constant over the study period (~ 1 month) for a given region, which defined a proportional relationship between infections and deaths. As a result, R_0_ remains comparable across regions reporting death counts consistently over time, even if the percentage of deaths reported across regions is variable (for example, if only deaths occurring in the hospital are reported in one region, compared to another where all deaths are accounted for). As a result, comparability in COVID-19 related deaths reporting standards was not necessary: the only pre-requisite was that death counts are reported using the same method in a given region over the period analysed.

### Data

#### Deaths from COVID-19

Regional level data on COVID-19 deaths were retrieved from data shared by national health ministries and/or public health agencies of Spain, Italy, Portugal, France, USA, and Canada (Table S1). Deaths were reported as daily new death counts or as a cumulative number.

#### Population and geographical data

Population structure by one- or five-year age groups, sex and region was retrieved from open access data shared by national institutes or administrations responsible for national statistics or demography (Table S1). The percentage of the region population aged ≥ 70 or ≥ 80 years was calculated in order to adjust for differences in way of life (e.g. rural regions have older population than metropolitan areas in Europe).

Region shapefiles by country were obtained from national geographical authorities, open source datasets or as provided in the coronavirus open data packages proposed by several national health agencies (Table S1). The region surface was either obtained from the geographical layer (land surface area), or calculated from the polygon extent. We estimated the percentage of each region surface with ≥ 5 inhabitants per km^2^ based on WorldPop 2020 raster dataset^23^. Population density was estimated as the population in the region divided by the surface after excluding areas < 5 inhabitants/km^2^ in order to limit the underestimation of population density when high heterogeneity existed between urban centers and sparsely populated territories within the same region (deserts, mountains, polar regions).

#### Weather/climate

Weather station reports since 1 January 2020 were obtained from US National Oceanic and Atmospheric Administration (NOAA) through the R-package {worldmet}. We extracted hourly temperature, relative humidity (RH), dew point temperature (DP), precipitation and windspeed observations for each station. Hourly absolute humidity (AH, in g/m^3^) was calculated from RH and temperature based on the Clausius-Clapeyron formula^24^. Daily minimum, maximum and mean values were calculated for temperature, AH, DP, as well as cumulative sum of precipitation and mean wind speed. Days with ≥ 5 missing hourly record (no observation of any parameter for > 20% of the day) were excluded to avoid biasing the period estimation with daily reports excluding night time temperatures.

Humidity is a key parameter for respiratory viruses, and was analysed using temperature-independent physical parameters (directly observed DP or calculated AH). Relative humidity (RH) was excluded from the analysis, since it is dependent on both temperature and AH through the Clausius Clapeyron formula, it would not characterize weather conditions specifically. Detailed properties of DP, AH and RH have been discussed extensively by Babin^21^.

Each region was attributed stations based on geographic location. All available observations in weather stations of the region contributed to the regional daily average. Weather stations can be located in mountains or sparsely populated locations where they record weather conditions that differ strongly from actual populated areas of the same region. To avoid this bias, observations were assigned population-based weights: we estimated the population located within 10 km of each weather station using WorldPop 2020 data; and for each day and each region, we calculated the total population within 10 km of any station reporting data for that day. Daily station observations were weighted in proportion of the population around each station relative to the total population. Stations located within 10-km of each other were included in a single buffer and each station was assigned equal weight within the buffer.

For each weather parameter, the regional summary value was calculated over the transmission period, during which infections were assumed to have occurred. We assumed that the transmission period had the same duration as the R_0_ calculation period, and occurred 3 weeks earlier according to the delay between infection and death (Figure S8).

Using this approach, the weather parameters averaged over the assumed transmission period and over each region included: minimum, mean and maximum average values for temperature, AH, DP, average cumulative precipitation per day and average wind speed.

#### Date of lockdown definition

Lockdown date was defined using Google mobility data as the date when a decrease > 25% in workplace localization was reported and sustained over 3 days in the region^25^. All references to a «date of lockdown» hereafter refer to this definition. This definition matched national lockdowns in European countries. This simplification was necessary due to the heterogeneity in social distancing measures taken at regional (state) level in the USA and Canada. The objective was to exclude periods where transmission would start to slow down due to these measures.

#### Distance to first region with 10 cumulative deaths

In order to account for the proximal spread of the epidemic, we considered that the first region affected in a country could represent a significant source of imported cases for other regions, and that the effect would decrease with the distance, reflecting spatial autocorrelation due to proximity in the spread of the epidemic. For each of the four European countries considered, we identified the first region with 10 cumulative deaths. In Portugal, two regions reached ≥ 10 deaths on the same day, and the region with the highest count (14 vs. 12) was selected. In the USA and Canada, the distance to the first region above 10 cumulative deaths was calculated separately for East Coast and West Coast, using the limit between Central and Mountain time zones. This was necessary due to the early start of the epidemic in the state of Washington, which reached 10 deaths by 2 March 2020, 16 days before the next state (New York on 18 March).

Within each country, the euclidean distance in kilometers between the main city in each region and the main city in the first region above 10 cumulative deaths was calculated.

This distance was a coarse but necessary simplification. Including more foci or accounting for actual passenger flux (rather than Euclidean distance) would rely on major assumptions requiring integration of complex data to describe connectivity between regions and/or importation rates. In addition, 10 cumulative deaths corresponded to a large number of clinical cases ensuring that by the time a region reached this level in the country, local case circulation was likely to exceed international case importation.

### Statistical methods

Statistical analyses were performed and figures (excepted maps) were produced using R version 4.0^26^. Maps were produced using ArcGIS 10.7.1 (ESRI, Redlands, CA).

### R_0_ estimation

Daily death counts were smoothed using a 5-day moving average filter to account for irregularities in data transmission and publication, which did not present a weekend effect. A 5-day span was a compromise between smoothing accidents or irregular reporting delays and flattening the slope which might lead to underestimations.

For each region, the exponential growth period was estimated using a log(deaths) = f(time) representation and r (the exponential growth rate) was extracted as the coefficient of a Poisson regression.

The exponential growth period was assumed to start when a linear increase was identified on the log(deaths) = f(time) representation for the region. A lower limit was set at the date when 10 cumulative deaths were reached. The end of the exponential growth period was defined as the date when incidence growth slowed down (indicated by a change in the slope of the log(deaths) = f(time) representation) in order to ensure that R0 was calculated over a period not impacted by the lockdown. If no inflection was identified, the end of the exponential growth period was set at maximum 28 day after lockdown, as explained below (see also Figure S8).

The lower boundary of 10 cumulative deaths was set to avoid early stochasticity and limitations in available recording of the first COVID-19 deaths at regional level. The upper boundary of 28 days after lockdown was defined to avoid the influence of lockdown measures on the growth rate of death counts, since we aimed to estimate SARS-CoV-2 R_0_ prior to implementation of major interventions. At individual level, the median delay between infection and death was 18 days, with a large interquartile range (IQR) of 9 to 24 days^27–33^. At regional level, we assumed that reported deaths corresponded to transmission events which had occurred in median three weeks earlier, and defined a 28-day boundary to cover the upper boundary of the IQR.

R_0_ was calculated for each region using the generation time method assuming a gamma distribution with parameters 7 and 5.2 for SARS-CoV-2 generation time^34,35^. In order to improve the adjustment of the regression, the start and end dates of the calculation period were allowed to shift by up to 2 days (+/− 1 day or + 1/+ 2 days for start date if the calculation period began at the date of 10 cumulative deaths) using the built-in function sensitivity.analysis() of the package {R0}^36^.

### Exclusion criteria

Regions not connected to mainland country (islands, Alaska…) were not included to avoid effects related to isolation.

Regions reporting less than 10 cumulative deaths from SARS-CoV-2 before lockdown + 28 days did not have a sufficient number of deaths to exclude early stochasticity and were not included. Regions where daily death counts remained low (< 5 deaths/day) were also not included, since a low number of deaths could be linked to either the absence of an epidemic start (only sporadic cases) or an R_0_ close to 1. Finally, regions with a smoothed daily death count < 10 deaths/day during the linear growth phase were excluded from the analysis. This avoided including regions where imported cases had so far failed to trigger an epidemic growth as “low risk” regions: as a result, only regions with a clearly established exponential growth phase were considered for this analysis (given current knowledge of the case-fatality ratio, 10 deaths correspond to at least 1000 cases).

One Italian region was excluded because there was no weather station data available except for 2 stations > 2000 m altitude.

### Analysis of the relationship between R_0_ and weather parameters

A directed acyclic graph (DAG) was constructed using Dagitty v3.0 web-based application (http://www.dagitty.net/dags.html) in order to visualise the relationships between R_0_ and explanatory covariates (Figure S2). Dependence and independence assumptions were verified using Spearman correlation coefficient. AH or DP are also strongly correlated to temperature^21^. The different weather covariates were observed during the estimated transmission period, which corresponded to the R_0_ calculation period lagged by 3 weeks to account for delay between infection and death. Weather covariates were included separately in the models. AH, DP and temperature were included as mean values over the period, but also minimum and maximum in order to account for the effect of extreme days. A generalized additive mixed model (gamm) regression was used to evaluate the effects of climate, population and other determinants on the value of R_0_, using a Gaussian distribution and the identity link function (package {mgcv}). A country-level random effect was included to account for within-country correlations. Canada presented with only two regions and was grouped with the USA for random effect, while the single region in Portugal was grouped with Spain. Significance threshold was set at 0.05. Models were compared using the percentage of deviance explained and Akaike’s information criterion (AIC). Univariate analyses were conducted assuming linear and non-linear effects, using B-splines to model non-linear effect of covariates. Linear approximation was found acceptable for log10(population density) since it minimized AIC while causing a minor loss in percentage deviance explained over splines. Non-linear effects were retained for weather variables and distance to the first focus (minimal AIC and large increase in percentage deviance explained for splines compared to linear).

We verified presence/absence of spatial autocorrelation in R_0_ values and in the final model residuals by calculating Moran’s I.

### Sensitivity analyses

First, we assessed the importance of the 3-week delay for weather variables (corresponding to the delay between transmission period and R_0_ calculation period based on death count exponential growth). For this, we tested a variety of lags from − 1 to 5 weeks from that estimated transmission period (i.e. 2–8 weeks from the R_0_ calculation period). The longer lags were likely irrelevant for actual transmission but aimed at identifying climate trends rather than weather influence. We included lagged weather variables as linear explanatory variables in the univariable hierarchical model or as non-linear explanatory variables in the multivariable hierarchical generalised additive model.

Second, we assessed the effect of the 28-day upper boundary to the exponential growth period by using a narrower window ending 18 days after date of lockdown. In this analysis, the exponential growth period ended at the date when incidence growth slowed down (indicated by a change in the slope of the log(deaths) = f(time) representation) or 18 days after lockdown if no inflexion was identified. We recalculated R_0_ for regions where the linear growth period retained for the main analysis extended beyond the 18-day limit, and followed the same plan as the main analysis.

Third, we assessed possible continent specific effects by fitting continent-specific splines for weather variables or for the distance to the first affected region in the final multivariate models.

## Supplementary Information

Supplementary Information 1.

Supplementary Information 2.

## Data Availability

All data used in this study is available from public data sources listed in Table S1. The only exception corresponded to early death counts at regional level in France which were obtained directly from the French Public Health Agency but have been publicly released since. The data table of regional-level values (R_0_ estimates, weather summary, population density etc.) used for the hierarchical generalized additive model analysis of the relationship between regional level R_0_ and weather covariates is provided in a supplementary csv file.

## Code availability

No custom code was used for this study beyond usual application of standard functions of softwares or R packages. Specific code sections wil be made available upon reasonable demand.

## References

[1] Jefferies S, French N, Gilkison C, Graham G, Hope V, Marshall J (2020). COVID-19 in New Zealand and the impact of the national response: A descriptive epidemiological study. Lancet Public Heal..

[2] Dighe A, Cattarino L, Cuomo-Dannenburg G, Skarp J, Imai N, Bhatia S (2020). Response to COVID-19 in South Korea and implications for lifting stringent interventions. BMC Med..

[3] Pollán M, Pérez-Gómez B, Pastor-Barriuso R, Oteo J, Hernán MA, Pérez-Olmeda M (2020). Prevalence of SARS-CoV-2 in Spain (ENE-COVID): A nationwide, population-based seroepidemiological study. Lancet.

[4] Lai C, Wang J, Hsueh P (2020). Population-based seroprevalence surveys of anti-SARS-CoV-2 antibody: An up-to-date review. Int. J. Infect. Dis..

[5] Dietz K (1993). The estimation of the basic reproduction number for infectious diseases. Stat. Methods Med. Res..

[6] Moriyama M, Hugentobler WJ, Iwasaki A (2020). Seasonality of respiratory viral infections. Annu. Rev. Virol..

[7] Matson MJ, Yinda CK, Seifert SN, Bushmaker T, Fischer RJ, Van DN (2020). Effect of environmental conditions on SARS-CoV-2 Stability in human nasal mucus and sputum. Emerg. Infect. Dis..

[8] Morris DH, Yinda KC, Gamble A, Fernando W, Huang Q, Bushmaker T (2021). Mechanistic theory predicts the effects of temperature and humidity on inactivation of SARS-CoV-2 and other enveloped viruses. Elife.

[9] Schuit M, Ratnesar-Shumate S, Yolitz J, Williams G, Weaver W, Green B (2020). Airborne SARS-CoV-2 is rapidly inactivated by simulated sunlight. J. Infect. Dis..

[10] Leung NHL (2021). Transmissibility and transmission of respiratory viruses. Nat. Rev. Microbiol..

[11] Briz-Redón Á, Serrano-Aroca Á (2020). The effect of climate on the spread of the COVID-19 pandemic: A review of findings, and statistical and modelling techniques. Prog. Phys. Geogr..

[12] Gillibert A, Jaureguiberry S, Hansmann Y, Argemi X, Landier J, Caumes E (2019). Comment on A. annua and A. afra infusions vs. Artesunate-amodiaquine (ASAQ) in treating Plasmodium falciparum malaria in a large scale, double blind, randomized clinical trial. Phytomedicine.

[13] Mecenas P, da Rosa Moreira Bastos RT, Rosário Vallinoto AC, Normando D (2020). Effects of temperature and humidity on the spread of COVID-19: A systematic review. PLoS ONE.

[14] Azuma K, Kagi N, Kim H, Hayashi M (2020). Impact of climate and ambient air pollution on the epidemic growth during COVID-19 outbreak in Japan. Environ. Res..

[15] Rubin D, Huang J, Fisher BT, Gasparrini A, Tam V, Song L (2020). Association of social distancing, population density, and temperature with the instantaneous reproduction number of SARS-CoV-2 in counties across the United States. JAMA Netw. Open.

[16] Merow C, Urban MC (2020). Seasonality and uncertainty in global COVID-19 growth rates. Proc. Natl. Acad. Sci. U S A..

[17] Baker RE, Yang W, Vecchi GA, Metcalf CJE, Grenfell BT (2020). Susceptible supply limits the role of climate in the early SARS-CoV-2 pandemic. Science (80-).

[18] Saad-roy CM, Wagner CE, Baker RE, Morris SE, Farrar J, Graham AL (2020). Immune life history, vaccination, and the dynamics of SARS-CoV-2 over the next 5 years. Science (80-).

[19] Zaitchik BF, Sweijd N, Shumake-Guillemot J, Morse A, Gordon C, Marty A (2020). A framework for research linking weather, climate and COVID-19. Nat. Commun..

[20] Warszawski, J., Bajos, N., Meyer, L., de Lamballerie, X., Seng, R., Beaumont, A.-L., et al. In May 2020, 4.5% of population in metropolitan France developped antibodies against SARS-CoV-2: First results from the national survey EpiCov [french] (2020). https://www.epicov.fr/wp-content/uploads/2020/10/Warszawski-et-al.-2020-Séroprévalence.pdf. Accessed 19 Jan 2021.

[21] Babin S (2020). Use of weather variables in SARS-CoV-2 transmission studies. Int. J. Infect. Dis..

[22] Gandini S, Rainisio M, Luisa M, Bellerba F, Cecconi F, Scorrano L (2021). A cross-sectional and prospective cohort study of the role of schools in the SARS-CoV-2 second wave in Italy. Lancet Reg. Heal Eur..

[23] Worldpop, Department of Geography and Geosciences University of Louisville, Department de Geographie Universite de Namur, CIESIN Columbia University. Global High Resolution Population Denominators Project (2018). https://www.worldpop.org/doi/10.5258/SOTON/WP00647. Accessed 25 Jun 2020.

[24] Lolli S, Chen YC, Wang SH, Vivone G (2020). Impact of meteorological conditions and air pollution on COVID-19 pandemic transmission in Italy. Sci. Rep..

[25] Google LLC. Google COVID-19 Community Mobility Reports (2020). https://www.google.com/covid19/mobility/. Accessed 4 Sep 2020.

[26] R Core Team. R: A language and environment for statistical computing. R Foundation for Statistical Computing, Vienna, Austria (2018). https://www.R-project.org/.

[27] Wiersinga WJ, Rhodes A, Cheng AC, Peacock SJ, Prescott HC (2020). Pathophysiology, transmission, diagnosis, and treatment of coronavirus disease 2019 (COVID-19): A Review. JAMA J. Am. Med. Assoc..

[28] Bi Q, Wu Y, Mei S, Ye C, Zou X, Zhang Z (2020). Epidemiology and transmission of COVID-19 in 391 cases and 1286 of their close contacts in Shenzhen, China: A retrospective cohort study. Lancet Infect. Dis..

[29] Richardson S, Hirsch JS, Narasimhan M, Crawford JM, McGinn T, Davidson KW (2020). Presenting characteristics, comorbidities, and outcomes among 5700 patients hospitalized with COVID-19 in the New York City Area. JAMA J. Am. Med. Assoc..

[30] Grasselli G, Greco M, Zanella A, Albano G, Antonelli M, Bellani G (2020). Risk factors associated with mortality among patients with COVID-19 in intensive care units in Lombardy, Italy Supplemental content. JAMA Intern. Med..

[31] ISCIII. Informe sobre la situación de COVID-19 en España (2020). https://www.isciii.es/QueHacemos/Servicios/VigilanciaSaludPublicaRENAVE/EnfermedadesTransmisibles/Documents/INFORMES/Informes COVID-19/Informe n^o^ 32. Situación de COVID-19 en España a 21 de mayo de 2020.pdf. Accessed 19 Jan 2021.

[32] Istituto Superiore di Sanità. Characteristics of SARS-CoV-2 patients dying in Italy Report based on available data on December 16th, 2020 (2020). https://www.epicentro.iss.it/en/coronavirus/bollettino/Report-COVID-2019_16_december_2020.pdf.

[33] Zhou F, Yu T, Du R, Fan G, Liu Y, Liu Z (2020). Clinical course and risk factors for mortality of adult inpatients with COVID-19 in Wuhan, China: A retrospective cohort study. Lancet.

[34] Salje H, Kiem C, Lefrancq N, Courtejoie N, Paireau J, Andronico A (2020). Estimating the burden of SARS-CoV-2 in France. Science.

[35] Wallinga J, Lipsitch M (2007). How generation intervals shape the relationship between growth rates and reproductive numbers. Proc. R. Soc. B Biol. Sci..

[36] Obadia T, Haneef R, Boëlle P (2012). The R0 package: A toolbox to estimate reproduction numbers for epidemic outbreaks. BMC Med. Inform. Decis. Mak..

[37] Gaudart J, Landier J, Huiart L, Legendre E, Lehot L, Bendiane M, Chiche L, Petitjean Aliette, Mosnier E, Kirakoya-Samadoulougou F, Demongeot J, Piarroux R, Rebaudet S (2021). Factors associated with the spatial heterogeneity of the first wave of COVID-19 in France: a nationwide geo-epidemiological study. The Lancet Public Health.

