# Supplementary material for: Cold and dry winter conditions are associated with greater SARS-CoV-2 transmission at regional level in western countries during the first epidemic wave
Source: Sci Rep. 2021 Jun 17;11:12756. doi: 10.1038/s41598-021-91798-9 (PMC8211690; doi:10.1038/s41598-021-91798-9)

Appendix

Cold and dry winter conditions are associated with greater SARS-CoV-2 transmission at regional level in western countries during the first epidemic wave.

Jordi Landier[1*], Juliette Paireau[2,3], Stanislas Rebaudet[1,4], Eva Legendre[1], Laurent Lehot[1], Arnaud Fontanet[5,6], Simon Cauchemez[2], Jean Gaudart[7]

[1] IRD, Aix Marseille Univ, INSERM, SESSTIM, Marseille, France.

[2] Mathematical Modelling of Infectious Diseases Unit, Institut Pasteur, UMR2000, CNRS, Paris, France.

[3] Santé publique France, French National Public Health Agency, Saint Maurice, France

[4] Hôpital Européen Marseille, France.

[5] Emerging Infectious Diseases Unit, Institut Pasteur, Paris, France.

[6] PACRI Unit, Conservatoire National des Arts et Métiers, Paris, France.

[7] Aix Marseille Univ, APHM, INSERM, IRD, SESSTIM, Hop Timone, BioSTIC, Marseille, France.

* corresponding author, jordi.landier@ird.fr

Systematic review of available evidence

Systematic reviews addressed the issue until 15 May 2020 (Briz-Redon A, et al, Prog Phy Geogr 2020 & Mecenas P et al, PLoS One, 2020).

Between 15 May 2020 and 15 December 2020, we searched PubMed database using the request: “(((COVID-19 OR SARS-COV-2)) AND (("2020/05/16"[Date - Publication]: "2020-12-15"[Date - Publication]))) AND (humidity [MeSH Terms] OR temperature [MeSH Terms] OR weather [MeSH Terms]OR climate[MeSH Terms])”. Of 464 references, we identified 82 research articles relative to the relationship between weather parameters and SARS-CoV-2 transmission. Of these, 67 (82%) analysed case, death or hospitalization counts, of which 11 presented the analysis of a single time series and 56 the analysis of cumulative or longitudinal counts in multiple locations ranging from regions to countries. Of 67, 52 (78%) presented only univariate analyses of weather variables, or the analysis of multiple correlated weather variables without evaluation of potential confounders. Only 15 (18%) studies analysed the growth rate or the reproduction number of SARS-CoV-2, of which only 4 studies did a multivariate analysis.

In spite of their large numbers, univariate studies and studies based on counts suffered from multiple biases and, according to systematic reviews, provided only low-grade evidence of a negative relationship between SARS-CoV-2 and temperature.

Table S1: Data sources

Country	Data type	source	source type	date accessed (or last update)	link
USA	deaths	The Covid Tracking Project	non governmental	29/09/2020	https://covidtracking.com/api/v1/states/daily.csv
USA	population	US Census Bureau	governmental	22/04/2020	https://www.census.gov/data/tables/time-series/demo/popest/2010s-state-detail.html
USA	shapefile	US Census Bureau	governmental	23/04/2020	https://www2.census.gov/geo/tiger/GENZ2018/shp/cb_2018_us_state_5m.zip
Canada	deaths	Public Health Infobase, Govt of Canada	governmental	15/05/2020	https://health-infobase.canada.ca/covid-19/
Canada	population	Statistics Canada	governmental	29/04/2020	https://www12.statcan.gc.ca/datasets/index-eng.cfm?Temporal=2016
Canada	shapefile	IGISMAP	non governmental	30/04/2020	https://map.igismap.com/share-map/export-layer/Canada_AL263/45fbc6d3e05ebd93369ce542e8f2322d
Italy	deaths	Protezione civile (Govt of Italy)	governmental	24/09/2020	https://raw.githubusercontent.com/pcm-dpc/COVID-19/master/dati-regioni/dpc-covid19-ita-regioni.csv
Italy	population	I.Stat	governmental	23/03/2020	http://dati.istat.it/Index.aspx?DataSetCode=DCIS_POPRES1
Italy	shapefile	I.Stat	governmental	23/04/2020	https://www.istat.it/storage/cartografia/confini_amministrativi/generalizzati/Limiti01012020_g.zip
Spain	deaths	Centro Nacional de Epidemiologia	governmental	25/05/2020	https://covid19.isciii.es/
Spain	population	Instituto Nacional d'Estadistica	governmental	20/04/2020	https://www.ine.es/jaxiT3/Datos.htm?t=31304#!tabs-mapa
Spain	shapefile	GADM	non governmental	20/04/2020	https://gadm.org/maps/ESP_1.html
Portugal	deaths	Data Science for Social Good Portugal	non governmental	21/10/2020	https://raw.githubusercontent.com/dssg-pt/covid19pt-data/master/data.csv
Portugal	population	Instituto Nacional d'Estadistica	governmental	21/04/2020	https://www.ine.pt/xportal/xmain?xpid=INE&xpgid=ine_indicadores&contecto=pi&indOcorrCod=0008273&selTab=tab0
Portugal	shapefile	Data Science for Social Good Portugal	non governmental	02/06/2020	https://github.com/dssg-pt/covid19pt-data/blob/master/extra/mapas/portugal/portugal.shp
France	deaths	Santé publique France/SI-VIC	governmental	24/04/2020	Not available in open access
France	population	INSEE	governmental	07/05/2020	https://www.insee.fr/fr/statistiques/1893198
France	shapefile	data.gouv.fr	governmental	02/06/2020	https://www.data.gouv.fr/fr/datasets/r/aacf9338-8944-4513-a7b9-4cd7c2db2fa9

Figure S1: Study regions in six countries of the North West hemisphere and selection criteria. Map generated using ArcGIS v10.7.1 (ESRI, Redlands, CA).

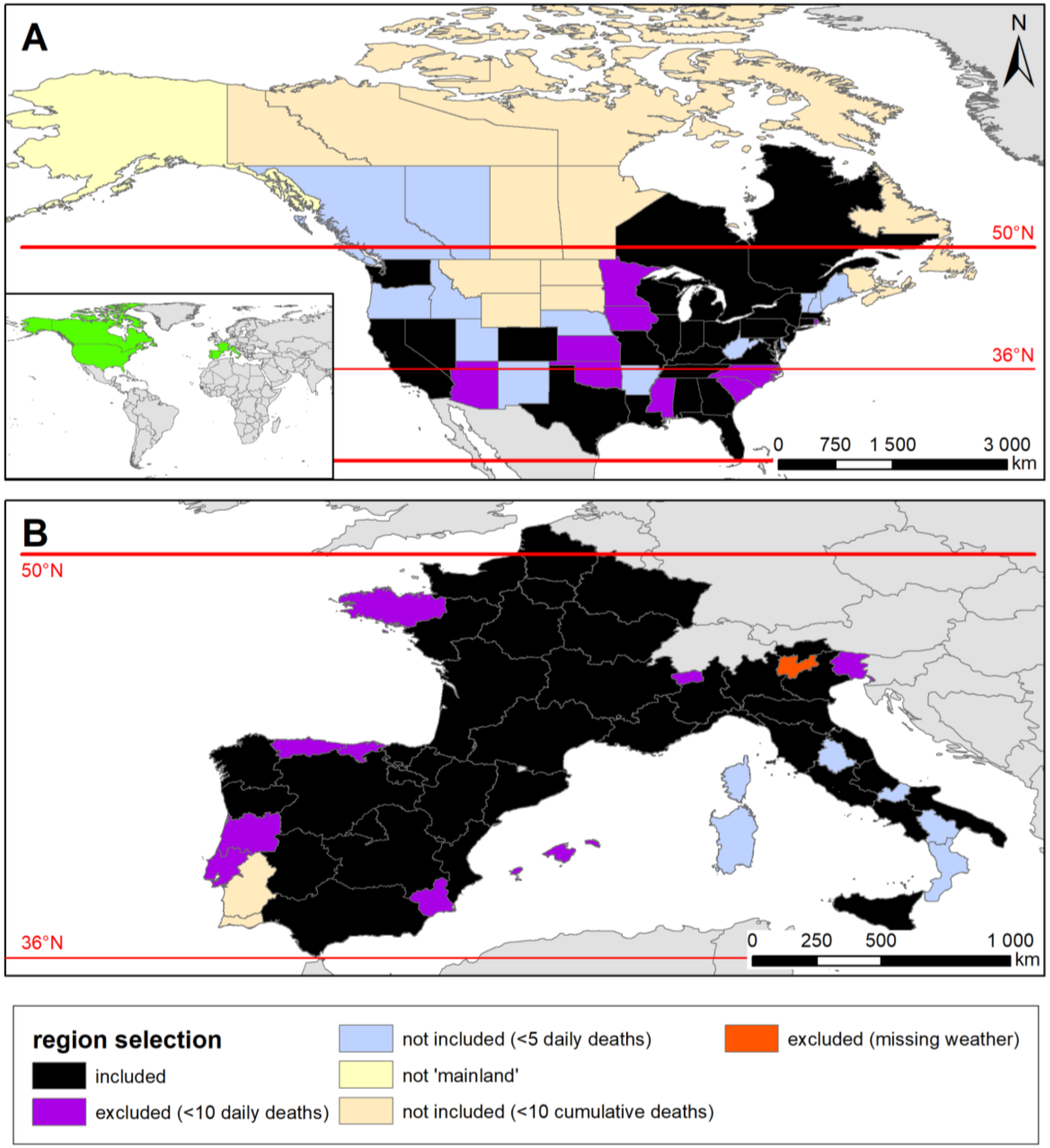

Figure S2: Directed acyclic graph for the relationship between weather/climate parameters and SARS-COV2 transmission as measured by R0. Graph generated using Dagitty v3.0 (http://www.dagitty.net/dags.html).

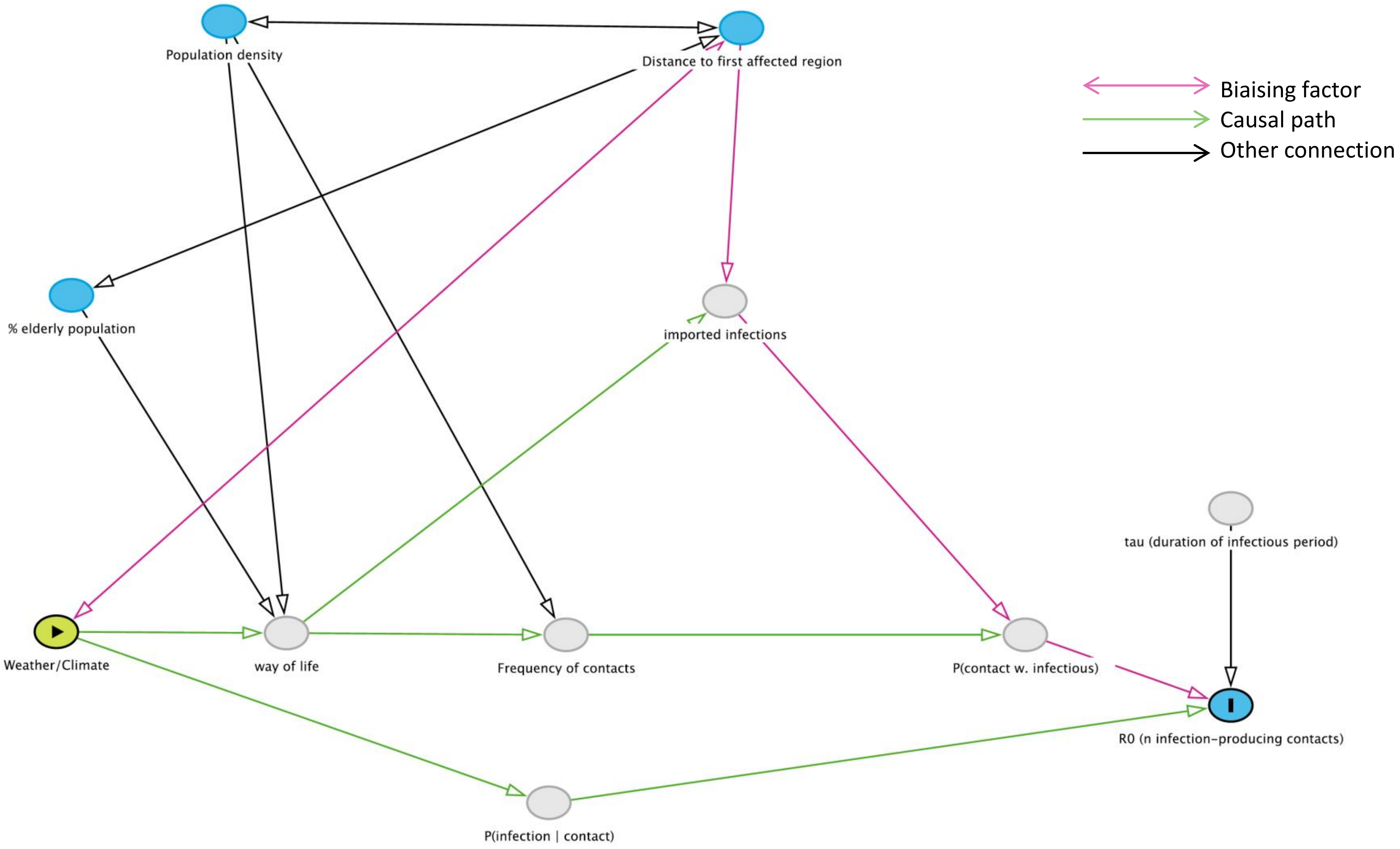

Figure S3: delay to peak in daily deaths according to the reduction of population mobility measured in transit stations (A) or in workplaces (B). The death count at the peak (maximal death count) is figured by the dot size (0, 100, 400).

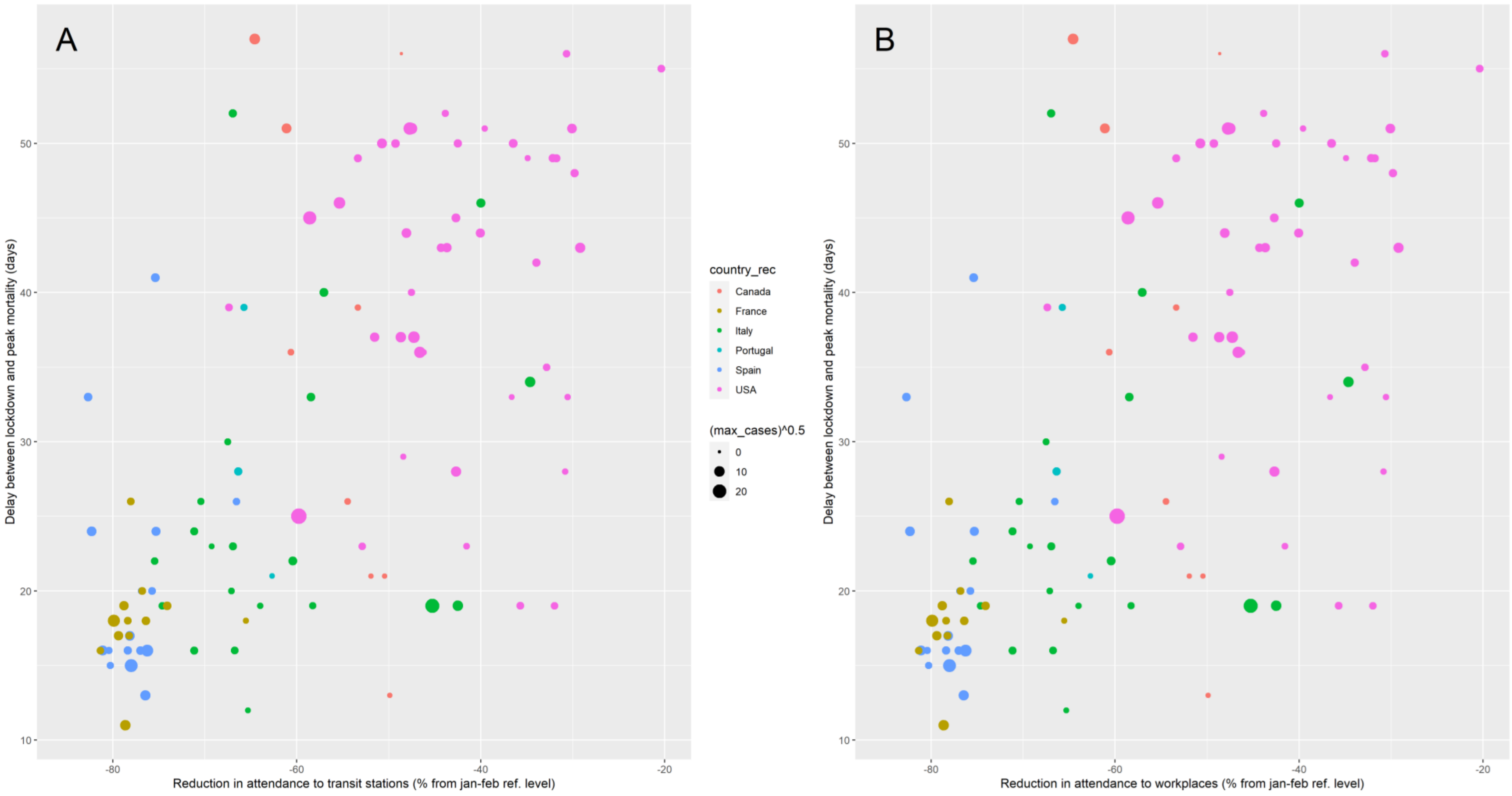

The correlation between the delay to reaching the peak in daily death counts and the reduction of population mobility was strong, with respective Spearman correlation coefficient values of 0.636 (p<10^-5) for transit stations and 0.631 (p<10^-5) for workplaces (p<10^-5).

Figure S4: R0 calculation periods by country

Vertical green lines indicate the window considered for R0 calculation, starting at the date of 10 cumulative deaths and ending 28 days after lockdown. Vertical blue line indicate the end of the window considered for R0 calculation in the sensitivity analysis, 18 days after lockdown. The portion of the epidemic curve defined as displaying linear trend on the log-scale and used to extract the growth rate and perform the R0 calculation is highlighted in red, and the corresponding linear regression is displayed in orange. (See Results section for median and IQR values for calculation periods).

France

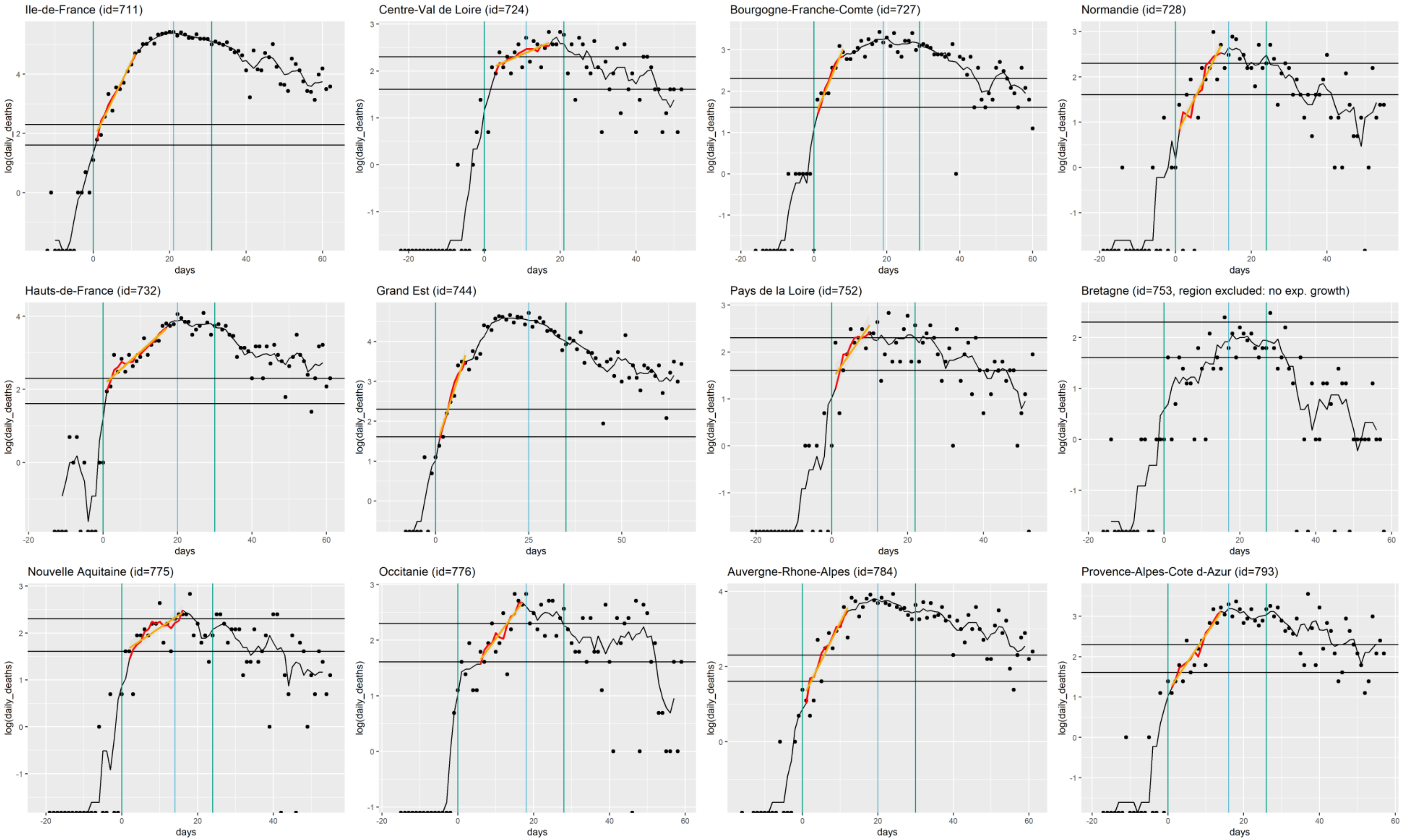

Ile-de-France (id=711)
Centre-Val de Loire (id=724)
Bourgogne-Franche-Comte (id=727)
Normandie (id=728)
Hauts-de-France (id=732)
Grand Est (id=744)
Pays de la Loire (id=752)
Bretagne (id=753, region excluded: no exp. growth)
Nouvelle Aquitaine (id=775)
Occitanie (id=776)
Auvergne-Rhone-Alpes (id=784)
Provence-Alpes-Cote d-Azur (id=793)
log(daily_deaths)
days

Italy

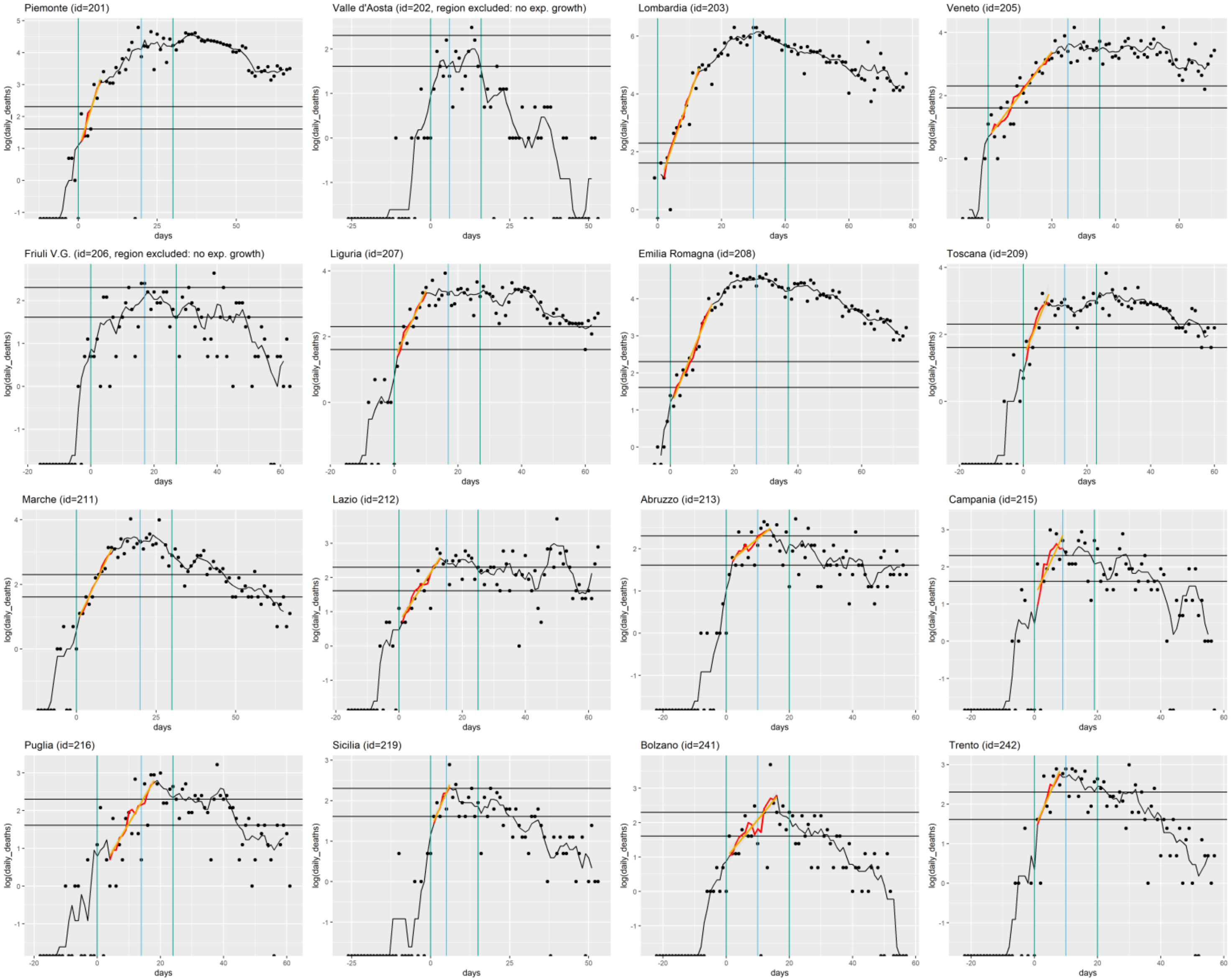

Piemonte (id=201)
Valle d'Aosta (id=202, region excluded: no exp. growth)
Lombardia (id=203)
Veneto (id=205)
Friuli V.G. (id=206, region excluded: no exp. growth)
Liguria (id=207)
Emilia Romagna (id=208)
Toscana (id=209)
Marche (id=211)
Lazio (id=212)
Abruzzo (id=213)
Campania (id=215)
Puglia (id=216)
Sicilia (id=219)
Bolzano (id=241)
Trento (id=242)
log(daily_deaths)
days

Spain

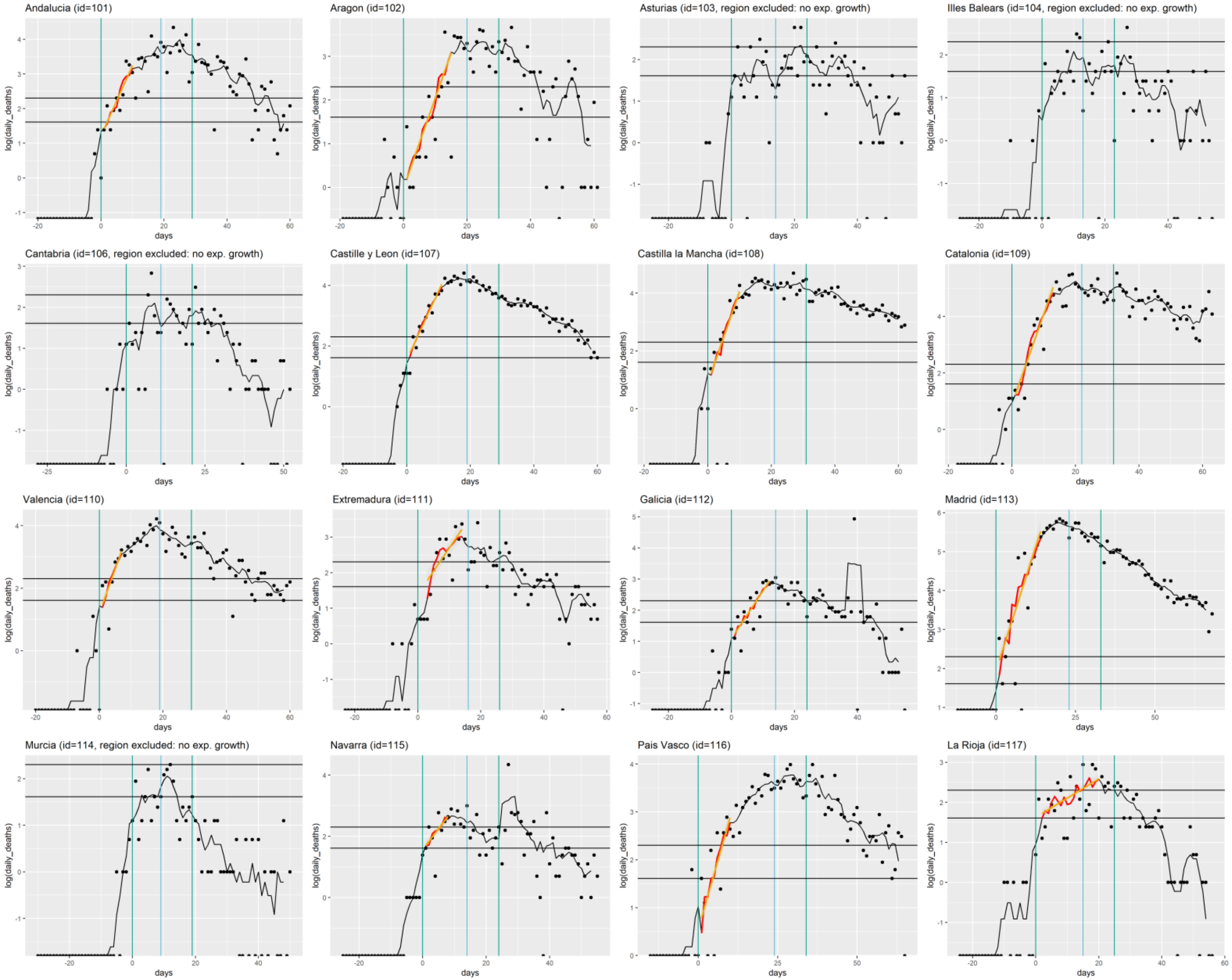

Andalucia (id=101)
Aragon (id=102)
Asturias (id=103, region excluded: no exp. growth)
Illes Balears (id=104, region excluded: no exp. growth)
Cantabria (id=106, region excluded: no exp. growth)
Castille y Leon (id=107)
Castilla la Mancha (id=108)
Catalonia (id=109)
Valencia (id=110)
Extremadura (id=111)
Galicia (id=112)
Madrid (id=113)
Murcia (id=114, region excluded: no exp. growth)
Navarra (id=115)
Pais Vasco (id=116)
La Rioja (id=117)
log(daily_deaths)
days

USA

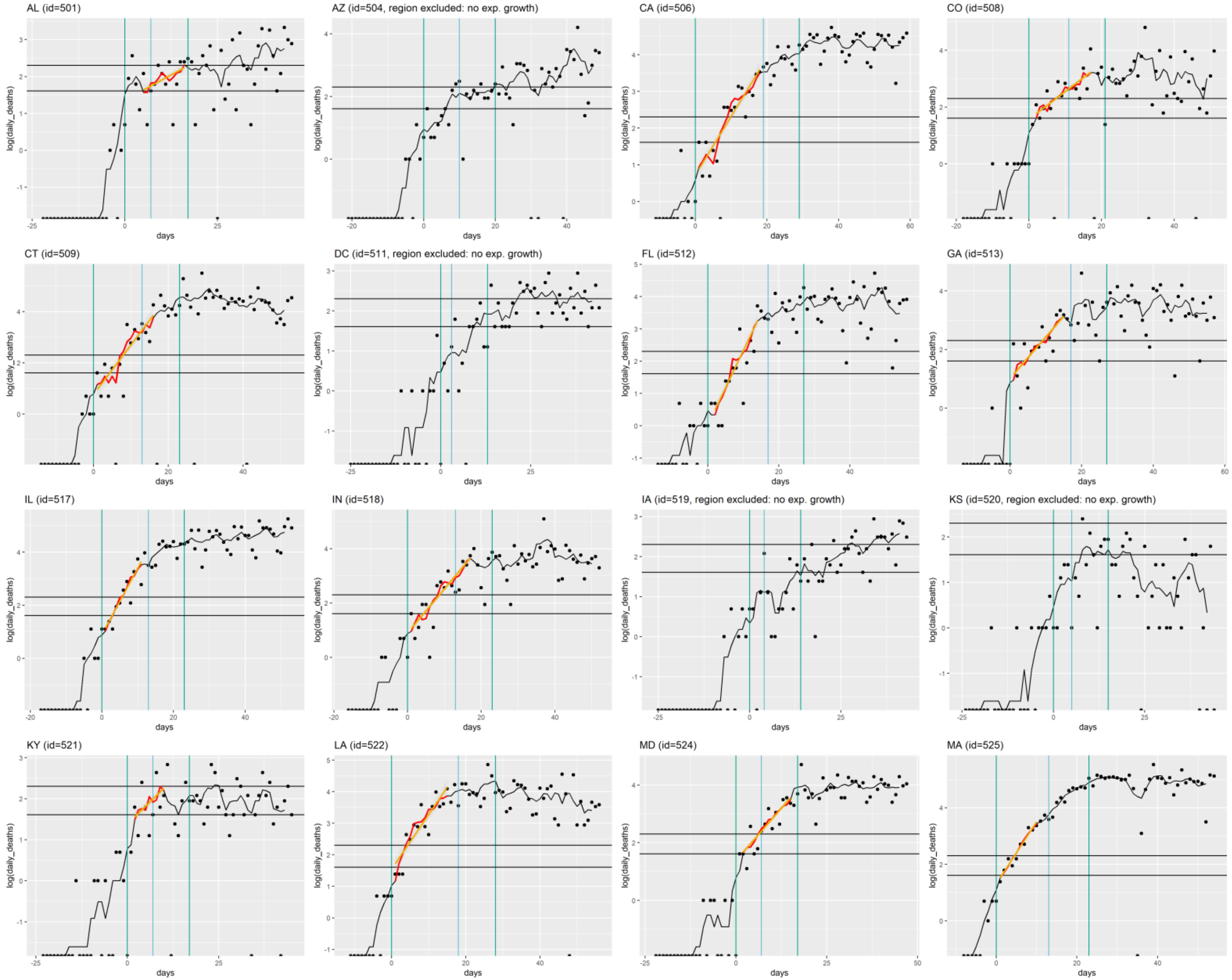

AL (id=501)
AZ (id=504, region excluded: no exp. growth)
CA (id=506)
CO (id=508)
CT (id=509)
DC (id=511, region excluded: no exp. growth)
FL (id=512)
GA (id=513)
IL (id=517)
IN (id=518)
IA (id=519, region excluded: no exp. growth)
KS (id=520, region excluded: no exp. growth)
KY (id=521)
LA (id=522)
MD (id=524)
MA (id=525)
days
log(daily_deaths)

USA (continued)

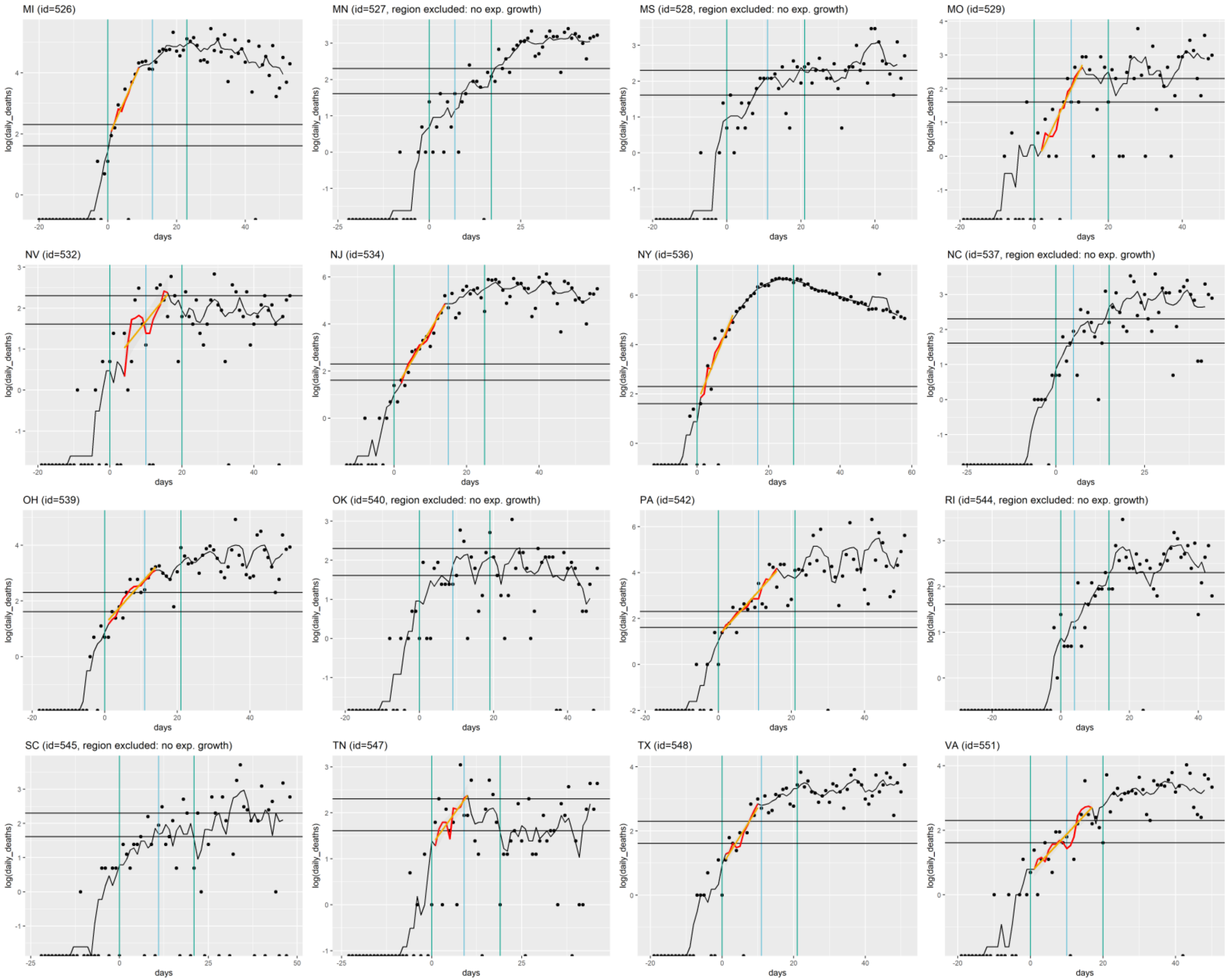

USA (continued 2), Portugal (303-305) and Canada (624,635)

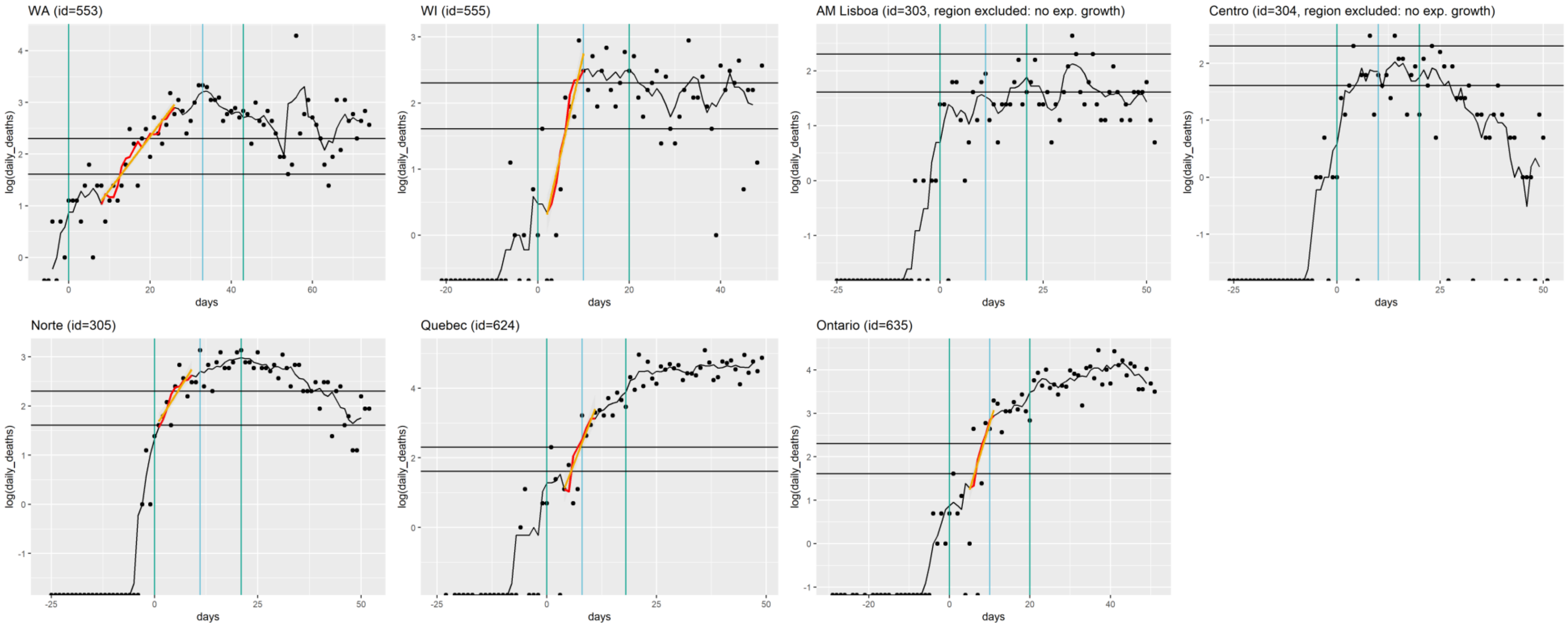

Table S2: Results of the univariable hierarchical model assuming a linear relationship between covariates and R0 value

Variable (linear)	estimate	95% confidence interval	p-value	AIC	Deviance explained (%)
Dew point temperature: min.	-0.075	[-0.115 - -0.035]	0.00049	146.22	19.02
Dew point temperature: mean	-0.079	[-0.122 - -0.037]	0.00053	146.40	18.80
Dew point temperature: max.	-0.081	[-0.127 - -0.034]	0.00123	148.00	16.55
Absolute humidity: min.	-0.185	[-0.295 - -0.074]	0.00175	148.61	14.95
Absolute humidity: mean	-0.162	[-0.26 - -0.065]	0.00187	148.74	14.77
Absolute humidity: max.	-0.140	[-0.23 - -0.051]	0.00319	149.76	13.38
Daily precip: mean	-0.092	[-0.152 - -0.031]	0.00411	150.24	12.72
Temperature: min.	-0.075	[-0.125 - -0.026]	0.00432	150.51	14.30
Temperature: mean	-0.072	[-0.123 - -0.022]	0.00653	151.47	15.09
dist. to 1st region affected	-0.050	[-0.089 - -0.01]	0.01586	152.85	12.38
Population density (log10)	0.808	[0.153 - 1.463]	0.01868	153.05	8.74
Temperature: max.	-0.056	[-0.104 - -0.008]	0.02606	154.11	12.28
Population density	0.001	[0 - 0.002]	0.05064	154.84	6.12
Windspeed: mean	-0.035	[-0.235 - 0.165]	0.73087	158.69	0.20
% pop over 80	0.021	[-0.103 - 0.145]	0.74377	158.68	1.17
% pop over 70	0.011	[-0.057 - 0.079]	0.74549	158.70	0.63

Table S3: Results of the univariable hierarchical model assuming a non-linear relationship between covariates and R0 value

Variables (B-spline)	p-value	Akaike Information Criterion (AIC)	Generalized Cross Validation (GCV)	Deviance explained (%)
DP: min.	0.0004762	146.2165	0.5785073	19.01842
DP: mean	0.0005223	146.3991	0.5801903	18.79512
Temp: mean	0.0041478	146.4266	0.5845332	26.75646
Daily precip: mean	0.0173321	146.8934	0.5935806	30.99777
dist. to 1st region affected	0.0071834	146.8959	0.5859508	21.37092
Absolute humidity: mean	0.0042660	147.0754	0.5879088	21.75236
Absolute humidity: min.	0.0046919	147.3259	0.5902896	21.51877
DP: max.	0.0012101	148.0024	0.5951022	16.54749
Temp: min.	0.0074311	148.0998	0.5978279	21.02545
Temp: max.	0.0094238	148.5818	0.6049323	24.27944
Absolute humidity: max.	0.0081602	148.8980	0.6045329	18.04753
% pop over 80	0.0248805	148.9918	0.6173410	31.38356
% pop over 70	0.4606301	157.5315	0.6956170	10.40253
Population density	0.1300157	154.6511	0.6623683	10.28745
Windspeed: mean	0.4369166	157.7554	0.7003610	13.15879

Based on the results of table S2 and S3, the log10 of population density was included as a linear covariate, due to a small gain in AIC with a limited loss in deviance explained. Distance to the first region affected was included as a spline to avoid issues in log transformation of 0 values (for regions first affected). Weather/climate parameters were included as non-linear predictors.

Table S4: Multivariable results for the relationship between R0 and weather parameters, under the assumption of a linear relationship obtained with the hierarchical generalized additive model. Weather parameters are temperature, absolute humidity, and dew point temperature, adjusted for distance to the first region affected, population density, and elderly population. The linear approximation appears valid for dew point temperature: the spline had a linear shape (Figure 6) and the linear approximation does not modify the percentage of deviance explained. The linear approximation results in a minor decrease in deviance explained for absolute humidity. It appears however not relevant for temperature since it results in a strong decrease in deviance explained.

Model	Variable	Estimate	95%CI	p-value
**Model 1b**	Intercept	1.48	[-0.34 - 3.3]	0.11618
	Population density (log10)	0.70	[0.06 - 1.34]	0.03562
	% population over 80	0.04	[-0.1 - 0.17]	0.61838
	Distance to first region affected in the country/coast	**spline**		0.0639
	**Mean temperature**	-0.08	[-0.13 - -0.02]	0.01163
	Dev. explained: 34.4%			
**Model 2b**	Intercept	2.36	[0.6 - 4.11]	0.01081
	Population density (log10)	0.47	[-0.14 - 1.08]	0.13580
	% population over 80	0.02	[-0.1 - 0.15]	0.72173
	Distance to first region affected in the country/coast	**spline**		0.0654
	**Mean AH**	-0.15	[-0.26 - -0.03]	0.01498
	Dev. explained: 32.0%			
**Model 3b**	Intercept	1.46	[-0.2 - 3.13]	0.09023
	Population density (log10)	0.49	[-0.12 - 1.1]	0.12005
	% population over 80	0.05	[-0.08 - 18]	0.47088
	Distance to first region affected in the country/coast	**spline**		0.0975
	**Mean Dew Point Temperature**	-0.07	[-0.12 - -0.02]	0.00498
	Dev. explained: 34.6%			

Figure S5: Sensitivity analysis of the link between weather covariates and R0 at different lags in a univariate model assuming a linear relationship.

Lag=0 (green line) indicates the model presented in Figure 5, lag=-1 indicates a weather summary period 7 days later, lag=1 indicates a weather summary period 7 days earlier…

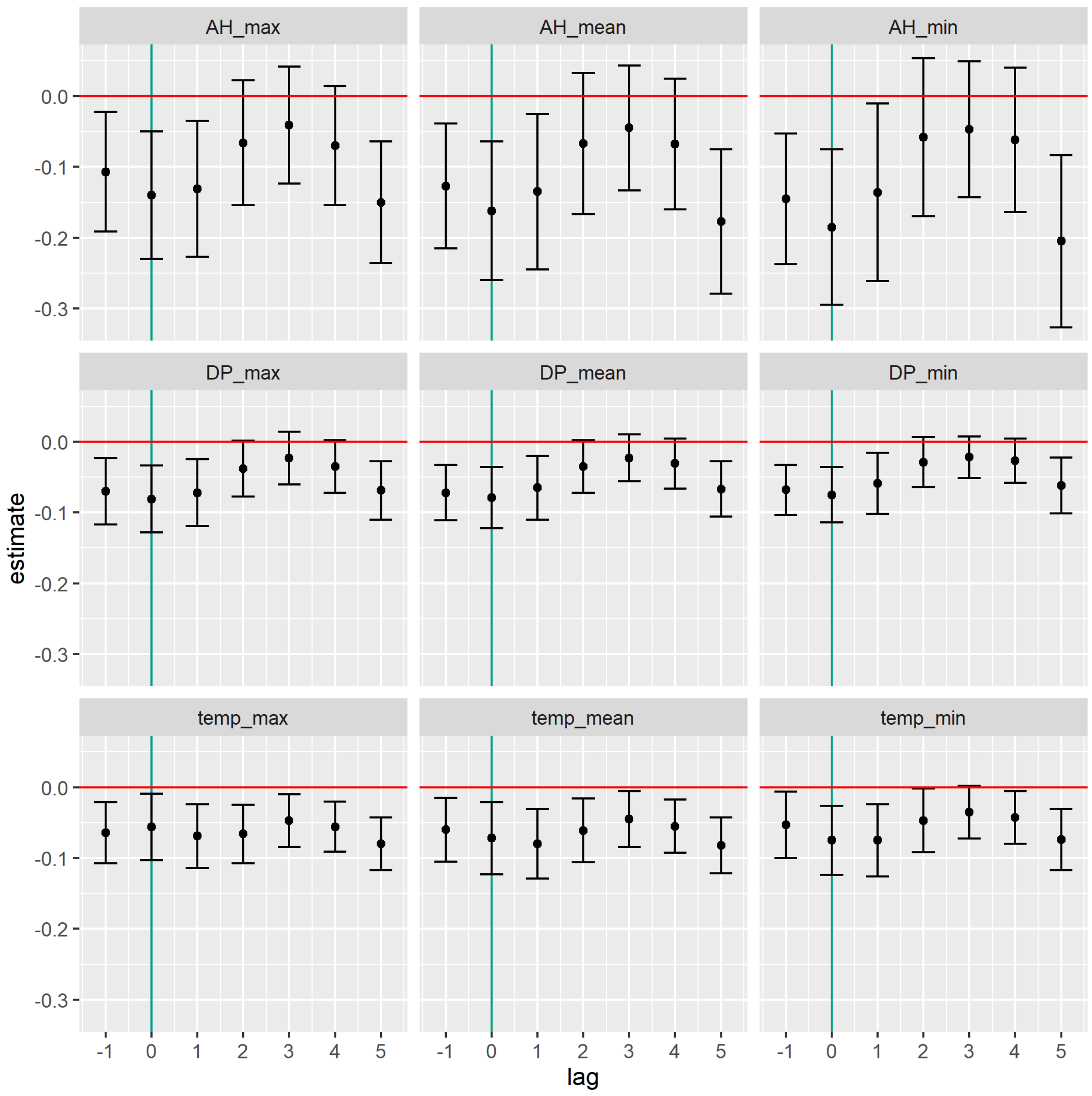

Figure S6

Sensitivity analysis for mean weather variable splines in the multivariate model.

Lag=0 indicates the final model, as shown in Figure 6, lag=-1 indicates a weather summary period 7 days later, lag=1 indicates a weather summary period 7 days earlier etc.

The effects of log10(population density) and percentage of population aged>80 were stable across lags.

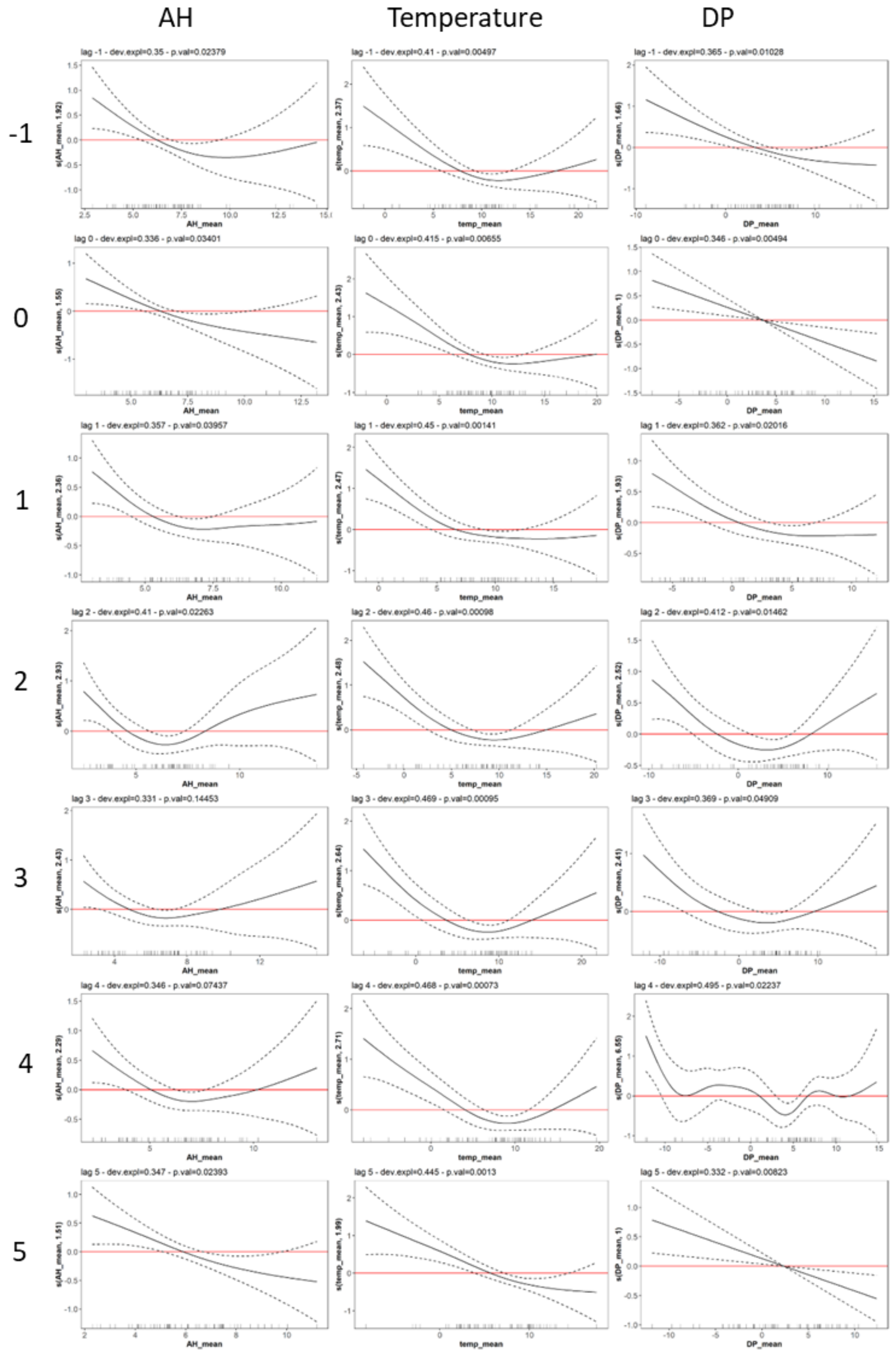

Figure S7: Spearman correlation coefficient between lagged weather variables used in sensitivity analysis

T: mean temperature, AH: mean absolute humidity, DP: mean dewpoint temperature

0: estimated transmission period (3-week lag from R0 calc. period); -1, 1-5: lagged observations

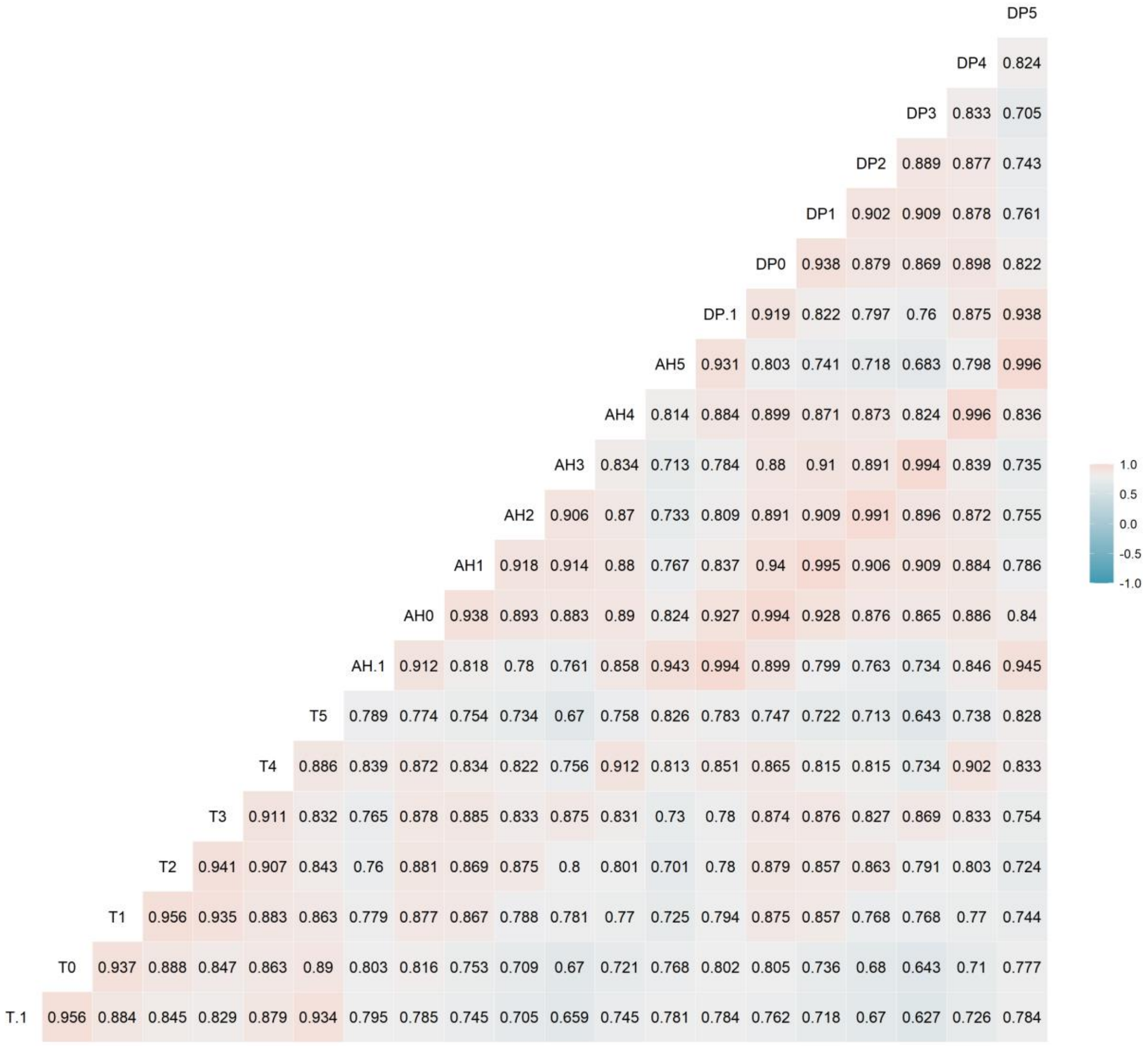

Figure S8: Schematic representation of time periods used in the study

The study period (green) was defined as the period where the growth of death counts was considered to occur without early stochasticity (cumulative death count>10) and with limited influence from lockdown (date≤lockdown+28 days).

Within the study period, the exponential growth period (in blue) was defined as the linear portion of the log(daily death count)=f(t) curve (black), and R0 was estimated on this period.

The transmission period (in red) was defined as the period when infections corresponding to the exponential growth period were acquired, i.e. 3 weeks earlier, over the same duration. Weather variable summary values were calculated over the transmission period.

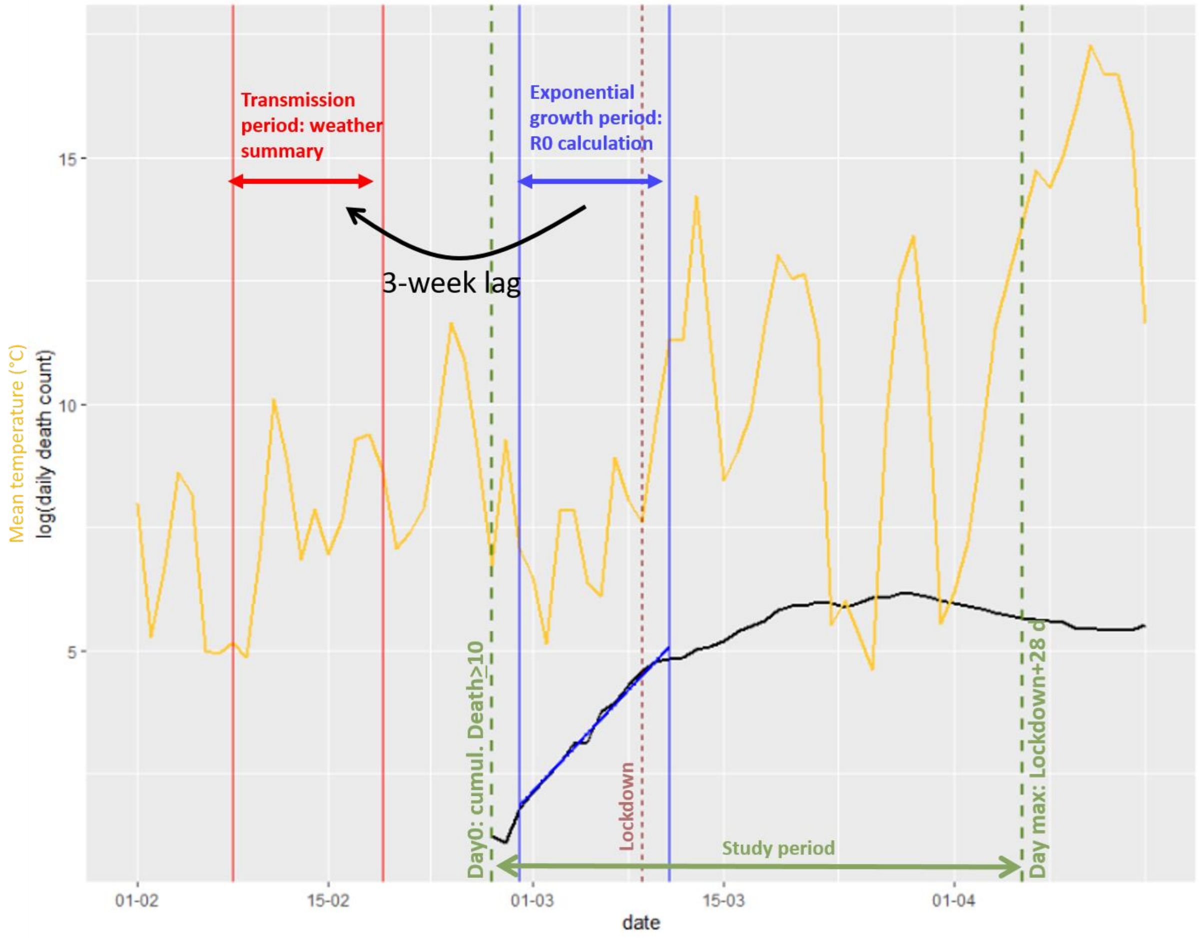

Figure S9: Experimental durations of virus survival according to absolute humidity (AH) converted from Morris DH et al, eLife 2021. We observe that the set of Temperature x Relative Humidity parameters actually describes a single AH gradient. The range of values associated with a decrease in virus survival matches the range of AH observed in meteorological parameters of our study.

**Experiment parameters**			
**Temperature***	**Relative humidity***	**Corresponding AH (g/m3)**	**Virus survival time (hours)***
10	40	**3.76**	**26.5**
10	65	**6.11**	**14.2**
10	85	**7.98**	**13.8**
22	40	**7.77**	**6.4**
22	65	**12.62**	**2.4**
22	85	**16.51**	**7.5**
27	40	**10.34**	**3.4**
27	65	**16.75**	**1.5**
27	85	**21.9**	**2.8**
*values extracted from Table 1, Morris et al, eLife 2021			

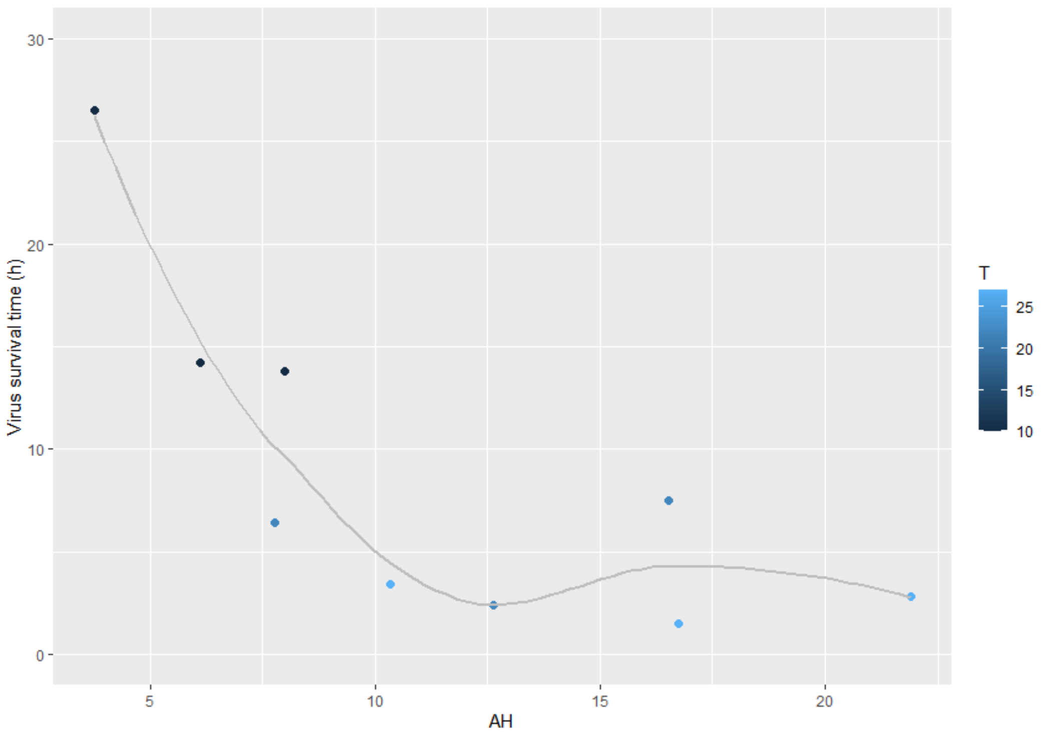

Supplement: Supplementary file 1 — Supplementary Information 1. [file 41598_2021_91798_MOESM1_ESM.pdf]
